# K562 erythroleukemia line as a possible reticulocyte source to culture *Plasmodium vivax* and its surrogates

**DOI:** 10.1016/j.exphem.2020.01.012

**Published:** 2020-02

**Authors:** Romy Kronstein-Wiedemann, Onny Klop, Jessica Thiel, Peter Milanov, Claudia Ruhland, Lars Vermaat, Clemens H.M. Kocken, Torsten Tonn, Erica M. Pasini

**Affiliations:** a Department of Experimental Transfusion Medicine, Medical Faculty Carl Gustav Carus, Technische, Universität Dresden, Dresden, Germany; b Biomedical Primate Research Centre, Rijswijk, The Netherlands; c Institute for Transfusion Medicine Dresden, German Red Cross Blood Donation Service North East, Dresden, Germany

## Abstract

Establishing an in vitro “red blood cell matrix” that would allow uninterrupted access to a stable, homogeneous reticulocyte population would facilitate the establishment of continuous, long-term in vitro *Plasmodium vivax* blood stage cultures. In this study, we have explored the suitability of the erythroleukemia K562 cell line as a continuous source of such reticulocytes and have investigated regulatory factors behind the terminal differentiation (and enucleation, in particular) of this cell line that can be used to drive the reticulocyte production process. The Duffy blood group antigen receptor (Fy), essential for *P. vivax* invasion, was stably introduced into K562 cells by lentiviral gene transfer. miRNA-26a-5p and miRNA-30a-5p were downregulated to promote erythroid differentiation and enucleation, resulting in a tenfold increase in the production of reticulocytes after stimulation with an induction cocktail compared with controls. Our results suggest an interplay in the mechanisms of action of miRNA-26a-5p and miRNA-30a-5p, which makes it necessary to downregulate both miRNAs to achieve a stable enucleation rate and Fy receptor expression. In the context of establishing *P. vivax*-permissive, stable, and reproducible reticulocytes, a higher enucleation rate may be desirable, which may be achieved by the targeting of further regulatory mechanisms in Fy-K562 cells; promoting the shift in hemoglobin production from fetal to adult may also be necessary. Despite the fact that K562 erythroleukemia cell lines are of neoplastic origin, this cell line offers a versatile model system to research the regulatory mechanisms underlying erythropoiesis.

## Highlights

• miR-26a and miR-30a knockdowns promote differentiation in Fy-transduced K562 cell lines.
• miR-26a and miR-30a knockdowns promote enucleation in Fy-transduced K562 cell lines.
• Data denote an interplay in the mode of action of miR-26a and miR-30a in erythropoiesis.
• *Plasmodium cynomolgi* and *P. knowlesi* invade, albeit inefficiently, Fy-transduced K562 cells.

The red blood cell (RBC) is the final stage of a journey that starts in the erythropoietic organs [1] with the maturation of hematopoietic stem cells. This multistep process involves the maturation of precursors committed to erythroid development to pro-erythroblasts, which evolve into erythroblasts. The maturation of progenitors takes place within designated multicellular clusters termed *erythroblastic islands* [2] and ultimately leads to reticulocytes that are released into the circulation, where they complete their maturation to erythrocytes. Erythropoiesis involves a complex array of biological processes characterized by a number of specialized mechanisms that are not fully understood [3]. The development of pro-erythroblasts into reticulocytes within the erythroblastic islands involves a number of interconnected steps that range from the attachment of erythroblasts to macrophages to nuclear reorganization (rearrangement, condensation, expulsion, and engulfment), the programmed destruction of organelles, and surface membrane protein sorting. Throughout erythroid maturation, surface markers as well as the cytoskeleton continuously evolve [4] because of rearrangements of the cell membrane and active exocytosis [5]. Rather than representing a single cell type, the term *reticulocytes* is used to describe a heterogeneous cell population comprising cells of different maturation levels (e.g., nascent reticulocytes or macroreticulocytes, siderocytes) [6]. Mammalian reticulocytes are characterized by the presence of polyribosomes, a variety of RNA species, and a small number of remnant mitochondria, and differ from reticulocytes from cold-blooded vertebrates (such as birds, reptiles, amphibians, and fish) by their lack of a nucleus. Although it has been suggested that one major evolutionary advantage provided by the absence of a nucleus may be their lack of susceptibility to viral infection [3], it is clear that both nucleated and nonnucleated reticulocytes are vulnerable to infections by parasites of the genus *Apicomplexa* such as *malaria* [7] and *babesia* [8].

In recent years the in vitro production of large numbers of reticulocytes has received renewed attention mainly because of the growing interest in blood farming and the renewed push for the establishment of continuous, long-term in vitro blood stage cultures for the reticulocyte-restricted human malaria parasite *Plasmodium vivax* in the wake of malaria eradication efforts [9]. Malaria is one of the most important human infectious diseases caused by *Plasmodium* parasites and affecting particularly the poorest populations living in the tropical and the subtropical areas of the world [10]. In general, the in vitro large-scale production of reproducible and stable reticulocytes carrying adult hemoglobin has proven to be cumbersome.

To tackle the need for a *P. vivax*-permissive, reproducible, and stable reticulocyte population and gain a better understanding of the regulatory factors behind the proliferation, differentiation, and enucleation of erythroid precursors, we set out to study the potential for differentiation of the erythroleukemia K562 cell line. We reasoned that this stable, continuously proliferating, immortal erythroleukemia line could provide the continuous source of reproducible and stable reticulocytes needed for long-term *P. vivax* in vitro culture. To obtain a *P. vivax*-permissive cell line, we introduced the Duffy receptor into a K562 mother line, as such receptor is known to be necessary for *P. vivax* invasion (Fy-K562) [11]. To promote more efficient enucleation in the Fy-K562 cell line we downregulated specific microRNAs (miRNAs).

MicroRNAs are short (20- to 23-nucleotide), endogenous, single-stranded RNA molecules that regulate posttranscriptional gene expression by translational repression or by destabilization of target transcripts [12,13]. MicroRNAs are important regulators of gene expression that control both physiological and pathological processes such as development and carcinogenesis. Recent reports indicate that specific miRNAs are involved in the regulation of proliferation, differentiation, and enucleation of red blood cell precursors [14]. In our present study, we found that the downregulation of miR-30a and miR-26a influences the ability of the Fy-K562 cell line to enucleate and drives the Fy-K562 toward erythroid differentiation. We observed a tenfold increase in the enucleation rate compared with the control, an increase in the production of hemoglobin, and expression of the erythroid marker CD71 coupled with a decrease in the lymphoid marker CD45. The Duffy receptor was stably expressed in Fy-K562 in which the miRs were downregulated, while in the control (no miR downregulation), the receptor disappeared after day 28. A 150-fold increase in α-globin after treatment with mithramycin A in the double knockdown was also noteworthy given that the α-globin increased only 50-fold in the control.

## Methods

Details of all methodologies, study approval/ethics are given in the Supplementary Material (online only, available at www.exphem.org).

### Cultivation of cell lines and plasmids and production of viral supernatants

Cultured human embryonic kidney 293T cells (DSMZ, Braunschweig, Germany) were used to produce lentiviral vectors for the transduction of mycoplasma-negative K562 cells with the Fy antigen and of Fy-K562 cells with pLV-[locker-miRNA] to downregulate specific miRNAs. Selection of single-cell clones of Duffy variant-expressing K562 cells and later miRNA double knockdown-transduced *Fy*-K562 cells was performed. The primer list for the generation of Duffy variants is provided in Supplementary Table E1 (online only, available at www.exphem.org).

### Duffy binding and invasion studies with *Plasmodium* species

Both isolated wild-type and transgenic fluorescent *P. knowlesi* and *P. cynomolgi* parasites [15] were used for binding and co-culture assays. In this set of experiments the K562 mother line not transduced with Duffy was taken along as a control. Binding assays were carried out either in suspension using a methodology adapted from Miller et al. [16] and Chitnis et al. [17] or using K562 monolayers; co-culture studies were carried out in suspension. Giemsa staining and counting under a light microscope were used as a detection method for wild-type parasites, while plates incubated with fluorescent parasites were assessed using an Operetta according to a methodology described before in Pasini et al. [18]. The experiment was repeated six times per variant for each parasite species; Fy variants were plated in quadruplicate.

### Induction of erythroid differentiation in Fy-K562 microRNA downregulated clones

Preliminary differentiation experiments were carried out to select suitable chemicals (Table 1) for further testing in the differentiation (35 days) of microRNA-downregulated Fy-K562 clones as part of an induction cocktail. Differentiation was carried out for 35 days in parallel both with and without macrophages in co-culture, as well as with and without chemical treatment (6 nmol/L mithramycin A) and the differentiation stage was evaluated every 7 days.

**Table 1:** All chemicals and concentrations tested in the differentiation of the Fy^b-long^-K562 erythroleukemia line*

Chemical	Concentration	Cell condition	CD45	CD71	CD235a	Fy	Hb production
Mithramycin	4 nmol/L	Healthy	+	=	+	=	Yes, high
	6 nmol/L	Healthy	–	++	++	=	Yes, high
	8 nmol/L	Healthy	–	++	++	=	Yes, high
Aphidicolin	100 nmol/L	Healthy	–	=	=	=	Yes, high
	200 nmol/L	Healthy	–	+	+	=	Yes, high
	300 nmol/L	Healthy	–	++	++	=	Yes, high
	400 nmol/L	Healthy	–	++	++	=	Yes, high
	500 nmol/L	Slow growth	–	++	++	=	Yes, medium
	1 µmol/L	Growth inhibition, cell debris	–	++	++	=	Yes, low
SAHA	1,5 µmol/L	Healthy	–	+	+	=	Yes, high
	2.0 µmol/L	Slow growth	–	+	+	=	Yes, high
	3.0 µmol/L	Growth inhibition, cell debris	–	+	+	=	Yes, low
Ara-C	100 nmol/L	Healthy	–	–	=	=	Yes, high
	250 nmol/L	Slow growth	–	–	=	=	Yes, high
	500 nmol/L	Growth inhibition, cell debris	–	–	=	=	Yes, low
Valproic Acid	500 µmol/L	Healthy	=	=	=	=	Yes, low
	1 mmol/L	Healthy	=	+	=	=	Yes, medium
	2 mmol/L	Growth inhibition, cell debris	=	+	+	=	Yes, medium
Resveratrol	1 µmol/L	Healthy	=	+	=	=	Yes, medium
	10 µmol/L	Slow growth	=	+	=	=	Yes, medium
	50 µmol/L	Growth inhibition, cell debris	=	+	=	=	Yes, medium
Hydroxyurea	340 µmol/L	Healthy	+	+	=	=	Yes, medium
	500 µmol/L	Healthy	+	+	=	=	Yes, medium
	750 µmol/L	Slow growth	+	+	=	=	Yes, medium
	1 mmol/L	Growth inhibition, cell debris	+	+	=	=	Yes, low
Nicotinamide	2.5 mmol/L	Healthy	–	–	–	=	No
	7.5 mmol/L	Slow growth	–	–	–	=	No
	10 mmol/L	Growth inhibition, cell debris	–	–	+	=	No
5-Azacytidine	1 µmol/L	Healthy	–	+	=	=	Yes, low
	2.5 µmol/L	Slow growth	–	+	+	=	Yes, low
	5 µmol/L	Growth inhibition, cell debris	–	+	+	=	Yes, low
Antimony(III) oxide	100 nmol/L	Healthy	=	=	=	=	Yes, low
	1 µmol/L	Slow growth	=	+	=	=	Yes, low
	10 µmol/L	Growth inhibition, cell debris	=	+	=	=	Yes, low
Vinblastine sulfate	10 nmol/L	Healthy	=	+	=	=	Yes, medium
	50 nmol/L	Healthy	+	+	=	=	Yes, low
	100 nmol/L	Growth inhibition, cell debris	+	+	=	=	Yes, low
Chromomycin A	1 nmol/L	Healthy	–	+	=	=	Yes, medium
	2.5 nmol/L	Slow growth	–	++	=	=	Yes, medium
	5 nmol/l	Growth inhibition, cell debris	–	++	=	=	Yes, low
Butyric acid	10 µmol/L	Healthy	=	=	=	=	Yes, low
	50 µmol/L	Healthy	=	+	=	=	Yes, low
	100 µmol/L	Growth inhibition, cell debris	–	+	=	=	Yes, low
Vanadium(V) oxide	100 nmol/L	Healthy	=	=	=	=	Yes, low
	1 µmol/L	Healthy	=	+	–	=	Yes, low
	10 µmol/L	Growth inhibition, cell debris	=	+	–	=	Yes, low
Aclacinomycin A	5 nmol/L	Healthy	–	+	=	=	Yes, medium
	25 nmol/L	Slow growth	–	++	+	=	Yes, medium
	50 nmol/L	Growth inhibition, cell debris	–	++	+	=	Yes, low
Idarubicin hydrochloride	1 nmol/L	Healthy	–	+	=	=	Yes, medium
	2.5 nmol/L	Slow growth	–	++	+	=	Yes, medium
	5 nmol/L	Growth inhibition, cell debris	–	++	+	=	Yes, low
Daunorubicin hydrochloride	1 nmol/L	Healthy	–	+	=	=	Yes, medium
	2.5 nmol/L	Slow growth	–	++	+	=	Yes, medium
	5 nmol/L	Growth inhibition, cell debris	–	++	+	=	Yes, low
Pirarubicin	1 nmol/L	Healthy	–	+	=	=	Yes, medium
	2.5 nmol/L	Slow growth	–	++	+	=	Yes, medium
	5 nmol/L	Growth inhibition, cell debris	–	++	+	=	Yes, low
Aloin	100 nmol/L	Healthy	–	+	=	=	Yes, medium
	300 nmol/L	Slow growth	–	++	+	=	Yes, medium
	500 nmol/L	Growth inhibition, cell debris	–	++	+	=	Yes, low

⁎ This table includes the read-out use for the assessment of differentiation, namely, hemoglobin production and surface marker (CD45, CD235a, CD71, and Fy) regulation. Hemoglobin production was measured by benzidine staining and classified as positive (Yes) or negative (No); when positive, an additional semiquantitative measure was given (low, medium, or high); surface markers were classified as downregulated (–), constant (=), upregulated (+), or very upregulated (++).

### Study approval and ethics

Informed consent was obtained from all donors, and donor materials were handled in line with the guidelines approved by the Ethics Committee of the Technical University of Dresden. All nonhuman primate infections were carried out in accordance with European and Dutch law after positive advice from the ethics committee (DEC).

### Statistical analysis

Data were analyzed in GraphPad Prism 5 using the Mann-Whitney *U* test. Values of less than 0.05 were considered significant. Data are presented as mean and error bars depict the SEM. No data were excluded from the analysis. No randomization or blinding was used in any of the experiments.

## Results

### Generation of Fy receptor-expressing K562 cell lines

**Figure 1:**
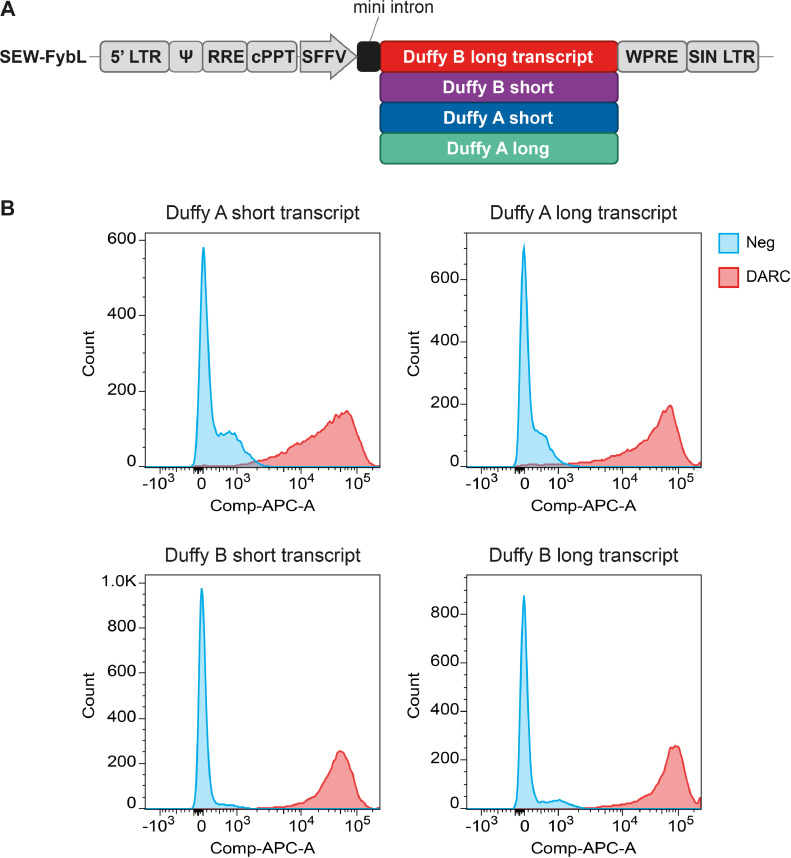
Generation of Fy-transduced K562 variants. **(A)** Schematic of the SEW-Fy vectors used to introduce the different Fy-variants into the K562 erythroleukemia cell line. **(B)** Confirmation by flow cytometry using an allophycocyanin (APC)-labeled anti-Fy antibody that the Fy receptor is expressed on the surface of all Fy-transduced K562 variants (*red*), not transduced control K562 cells (*blue*).

It is well known that *P. vivax* [19] and its closely related non-human primate malarias *P. knowlesi* [20] and *P. cynomolgi* [21] require the Duffy blood group antigen receptor to invade their host red blood cells. However, the K562 erythroleukemia cell line does not express this antigen. Therefore, the stable introduction of this receptor into K562 cells was achieved by retroviral transduction. The Duffy blood group system comprises two antigens, which differ in an amino acid substitution (G>D) at position 44 causing major polymorphisms (Fy^a^ or Fy^b^). Two transcription variants are known for Fy^a^ or Fy^b^ differing in the 5′ UTR and coding sequence, where isoform “short” has a shorter and distinct N-terminus compared with isoform “long.” As parasite binding and thus invasion may vary depending on the specific Duffy variant, different Duffy alleles, alone and in combination, have been transduced into K562 cells, resulting in the generation of five different Fy blood group-expressing K562 cell lines (Fy^a-sho^rt; Fy^a-long^; Fy^b-sho^rt; Fy^b-long^ and Fy^a/b long/long^) (Figure 1A).

For the malaria parasite to invade the target blood cells, it is important for the Fy receptor to be stably expressed and exported to the surface of the K562 erythroleukemia cells lines, which were generated. By use of flow cytometric analysis and anti-Fy antibodies, the surface location of the Fy receptor and its availability to the parasite were confirmed (Figure 1B).

### Binding and invasion of *P. cynomolgi* and *P. knowlesi* to Fy-transduced K562 cells

**Figure 2:**
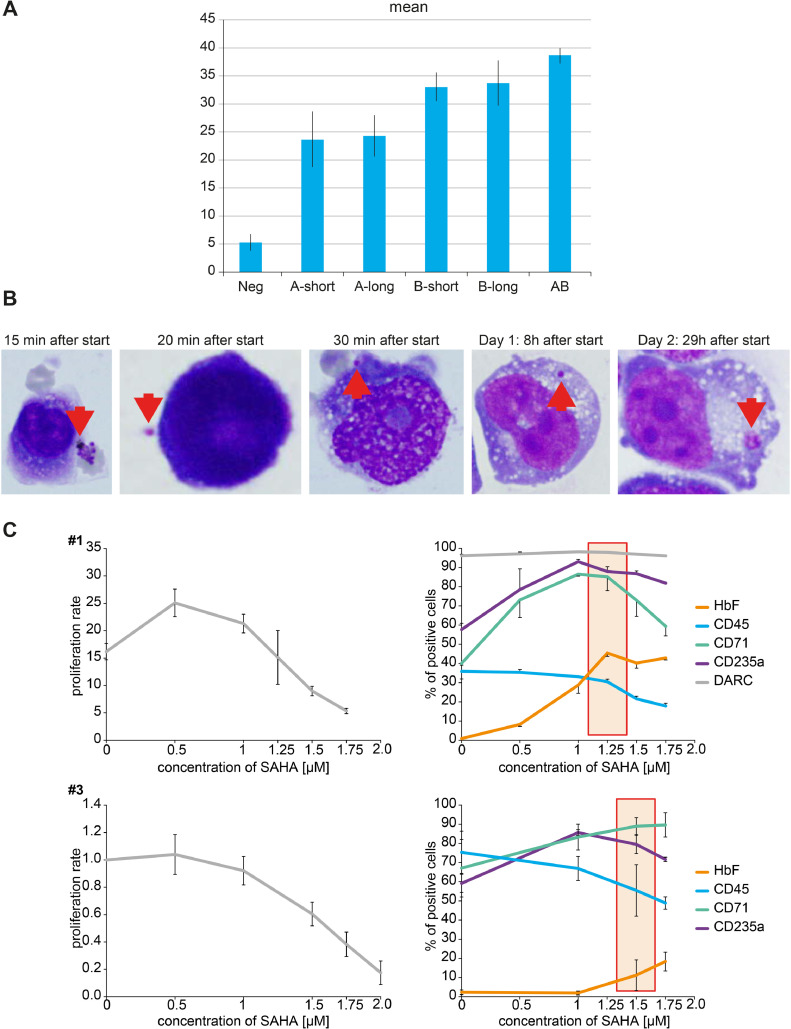
Selection of Fy-transduced K562 variants. **(A)** Results of the binding experiments with the different Fy-transduced K562 variants using fixed K562 monolayers and free *P. cynomolgi* merozoites (one representative experiment of six is shown), each Fy variant was plated in quadruplicates (*vertical bars* indicate standard deviations). **(B)** Invasion of *P. cynomolgi* merozoites into the Fy^b-long^-K562 variant; the *red arrows* indicate the different moments of parasite binding (at 20 and 30 min) and invasion/growth inside the cell (at 8 and 29 h). **(C)** The response to the induction of differentiation of Fy^b-long^-transduced K562 cells using SAHA is clone (Nos. 1 and 2) dependent. *Neg*=non-transduced control K562 cells.

To select for the variant of Fy-expressing K562 that gave rise to the best parasite binding, a number of binding assays were performed both in solution and using K562 monolayers with wild-type and transgenic fluorescent *P. knowlesi* and *P. cynomolgi*. In these experiments, the K562 mother line that is not expressing the Fy receptor was used as a control. Binding experiments in suspension with schizont-derived merozoites were repeated several times, but always resulted in a high standard deviation, likely because the rupture of schizont-infected erythrocytes is seldom synchronous. Increased reliability was obtained by using isolated merozoites to perform the binding studies both in solution and using Fy-K562 monolayers, where K562 monolayers gave superior, more reproducible results (Figure 2A). No binding to cells of the K562 mother line was observed. By use of enzyme-linked immunosorbent assay (ELISA), the Fy receptor was found to be stable and intact after fixation. Based on the binding studies, the Fy^b-long^-expressing K562 cell line was selected for further evaluation.

Some of the cultures used for the binding studies were followed over a longer period to assess if invasion would follow binding. Again, the K562 mother line that is not expressing the Fy receptor was used as a control. Samples were taken at different time points, and limited invasion but not growth past the 29 hours by *P. cynomolgi* parasites was observed (Figure 2B). No invasion was observed in the control cells.

### Response of Fy^b-long^-transduced K562 cells to the induction of differentiation using chemicals

As malaria parasites are dependent on hemoglobin for their growth and, thus, mature only inside cells of the erythrocyte lineage, Fy^b-long^-transduced K562 cell clones that showed the most tendency to develop along the erythroid lineage were selected for differentiation (Supplementary Figure E1, online only, available at www.exphem.org). The response to the induction of differentiation of Fy^b-long^-transduced K562 cells using chemicals is clone dependent (Figure 2C). A number of different chemicals (Table 1) were used, which according to the literature bring about K562 terminal differentiation 22, 23, 24, 25. At least three different concentrations (high, medium, low) of 24 chemicals were tested for their ability to induce Fy^b-long^**-**K562 differentiation (Table 1). A few promising chemicals (able to induce high hemoglobin production and increased levels of differentiation into erythroblasts) were selected for further testing: suberoylanilide hydroxamic acid (SAHA, 1.5 µmol/L), which was the first agent found to be active when we performed the screening; cytarabine (Ara-C, 100 nmol/L); mithramycin (6 nmol/L); aphidicolin (400 nmol/L). In the following experiments, we consistently used mithramycin as aphidicolin and Ara-C were found to be toxic over a 35-day period and SAHA's induction was found to be weaker. However, enucleation to reticulocytes is low and remains low even after an attempt to reproduce the unique environment characteristic of the erythroblastic island by adding macrophages.

### MicroRNA knockdown in Fy^b-long^-K562 cells

In an attempt to induce terminal erythroid differentiation and obtain higher levels of enucleation, miR-26a-5p and miR-30a-5p were knocked down by transducing Fy^b-long^-K562 cells with a locker plasmid against miR-26a-5p and/or miR-30a-5p. This resulted in two single knockdown cell lines (Fy^b-long^-K562-L-26a and Fy^b-long^-K562-L-30a) in which either miR-26a-5p or miR-30a-5p was knocked down, respectively, and a double-knockdown cell line named Fy^b-long^-K562-L-26a-30a. Single-cell clones of the double-knockdown cell line Fy^b-long^-K562-L-26a-30a were obtained, and clone 26 (C26) was characterized by a number of downstream analyses. As a control, Fy^b-long^-K562 cells were transduced with the empty locker vector.

### Effect of double knockdown on hemoglobin production during induction of erythroid differentiation

**Figure 3:**
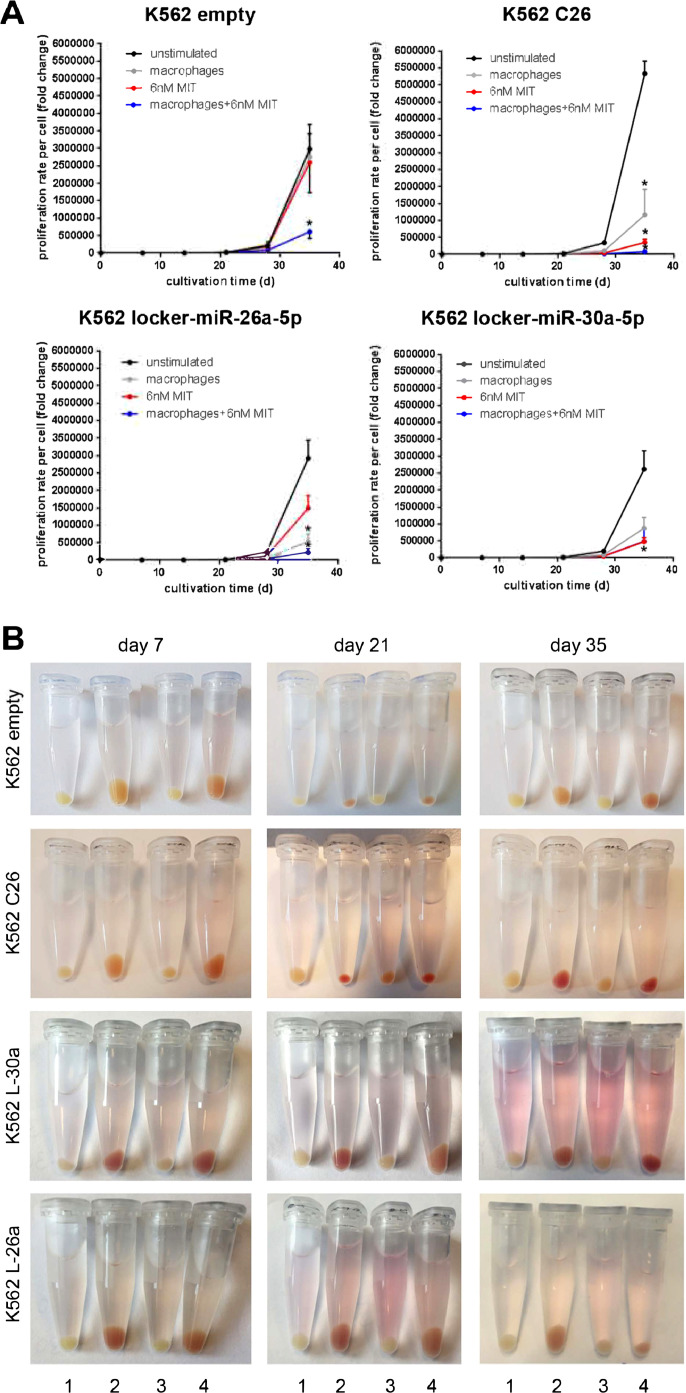
Effect of miRNA downregulation on the proliferation rate and production of hemoglobin of Fy-K562. Fy^b-long^K562 cells were transduced with the empty locker vector, the locker-miR-26a-5p and the locker miR-30a-5p, the locker-miR-26a-5p or the locker miR-30a-5p. **(A)** The proliferation rate of these different Fy^b-long^K562 cell lines are compared under four different conditions: untreated (*black solid line*), treated with macrophages (*gray solid line*), treated with 6 nmol/L MIT (*red solid line*), and treated with macrophages and 6 nmol/L MIT (*blue solid line*). Data are presented as means ± SEM. Tests were performed two-sided. *p < 0.05 (Mann–Whitney *U* test). *N* = 4 independent experiments. **(B)** K562 cell pellets at different time points of differentiation under four different conditions: untreated (1), treated with 6 nmol/L MIT (2), treated with macrophages (3), and treated with macrophages and 6 nmol/L MIT (4). One of four representative experiments is shown.

**Figure 4:**
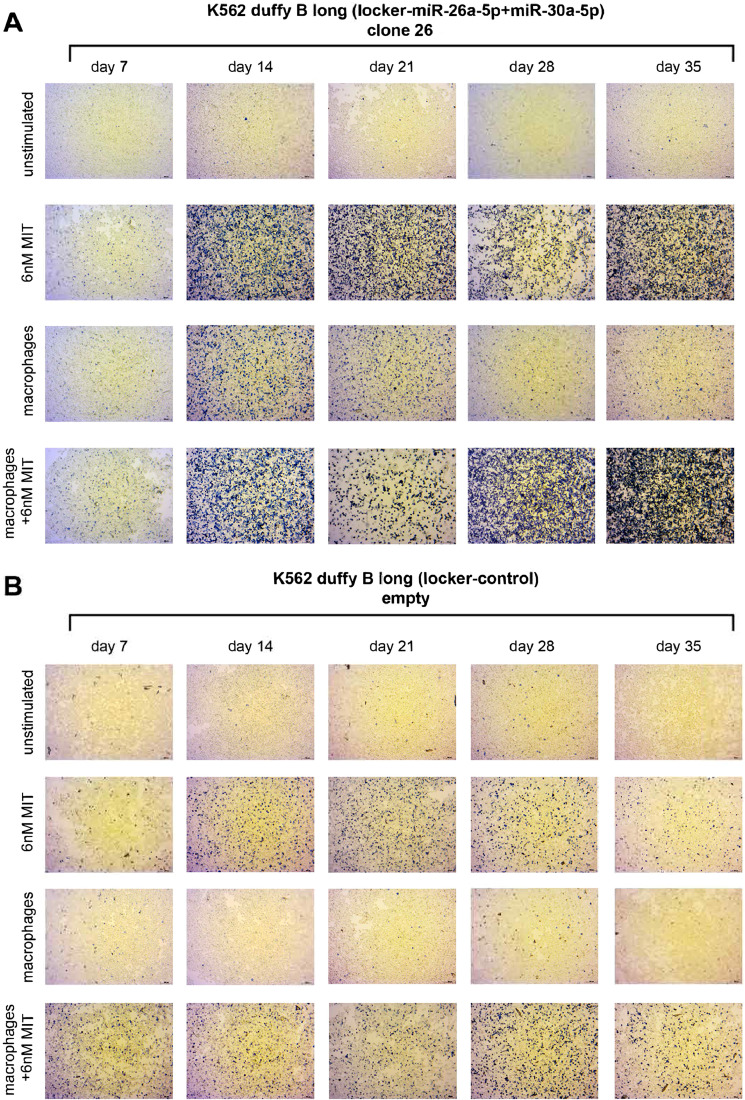
Effect of miRNA downregulation on the production of hemoglobin by benzidine staining. **(A,B)** Fy^b-long^K562 cells either double transduced with the locker miR-26a-5p and the locker miR-30a-5p vectors **(A)** or transduced with the empty locker control vector **(B)** were cultured under four different conditions for 35 days. Every 7 days, 3 × 10^5^ cells were stained with benzidine, and cytospins were performed. Bar = 20 µm. One of four representative experiments is shown.

C26 showed the highest proliferation, as measured by cell counting with Trypan Blue, in the untreated stage as compared with Fy^b-long^-K562-L-26a and Fy^b-long^-K562-L-30a (Figure 3A). Two inducers of erythroid differentiation—mithramycin A (MIT), which in our hands was the most promising differentiation-inducing chemical, and/or macrophages to mimic maturation in erythroblastic islands—were added to promote differentiation. Treatment of cells with MIT and/or co-culture with macrophages led to a reduction of proliferation and the promotion of erythropoiesis in all cell lines (Figure 3A). Viability studies show that the reduction in proliferation and promotion of erythropoiesis is not due to a toxic effect of MIT (Supplementary Figure E2, online only, available at www.exphem.org). We monitored the quantity of hemoglobin formation by the intensity of the red coloration of the cell pellets (Figure 3B) and confirmed hemoglobin production using benzidine staining (Figure 4; Supplementary Figure E3, online only, available at www.exphem.org). While the pellets of the control cells show only a slight reddish color, we observed a marked increase in hemoglobin formation in the two single-knockdown cell lines. However, this hemoglobin production seems to be transient in both the Fy^b-long^-K562-L-26a and Fy^b-long^-K562-L-30a cell lines, with maximum hemoglobin formation at days 21–28, as confirmed by benzidine staining (Supplementary Figure E3, online only, available at www.exphem.org). Only the C26 cells showed strong hemoglobin production until day 35 (Figure 4A), while the empty locker vector transduced Fy^b-long^-K562 cells showed only low levels of hemoglobin production (Figure 4B).

Furthermore, co-culture of the C26 with macrophages also led to transient hemoglobin production in the absence of MIT treatment, the hemoglobin disappearing by day 35.

**Figure 5:**
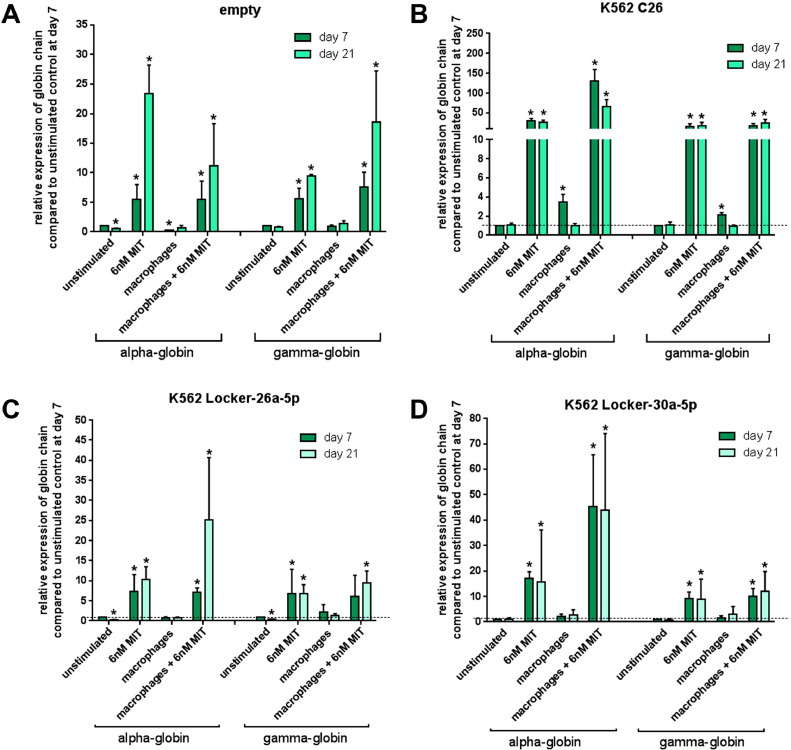
Effect of miRNA down regulation on the mRNA expression levels of hemoglobin chains. (**A–D**) Real-time PCR expression analysis of α- and γ-globin chain mRNA in Fy^b-long^K562 cells transduced with the empty locker vector **(A)**, locker-miR-26a-5p and locker miR-30a-5p (C26) **(B)**, locker-miR-26a-5p **(C),** or locker miR-30a-5p **(D)** vector. Cells were cultured under four conditions. Relative fold changes in expression (normalized to GAPDH) were calculated by the ΔΔ*CT* method and values are expressed as 2^–ΔΔ^*^CT^* (*n* = 4 independent experiments). Data are presented as means ± SEM. Tests were performed two-sided. *p < 0.05 (Mann–Whitney *U* test).

To acquire information on the possible relationships between hemoglobin induction and globin gene expression, we quantified the expression of α- and γ-globin mRNA after treatment of double-transduced Fy^b-long^-K562 cells with MIT in the presence or absence of macrophages. The β-globin gene was not considered because Fy^b-long^-K562 cells express very low levels of this gene [26]. All cell lines showed an increase in α- and γ-globin mRNA expression after treatment with MIT (Figure 5), with the highest yield of both globin chains in the double-transduced cells (α-globin: 150-fold vs. 25- to 80-fold; γ-globin: 50-fold vs. 10- to 25 fold). Additionally, macrophages seem to promote only α-globin mRNA expression, whereas γ-globin mRNA expression seems to be unaffected (Figure 5). The results suggest that only MIT, and not the co-culture with macrophages, provides hemoglobinization.

### Effect of double knockdown on expression of erythroid-specific surface markers during induction of erythroid differentiation

**Figure 6:**
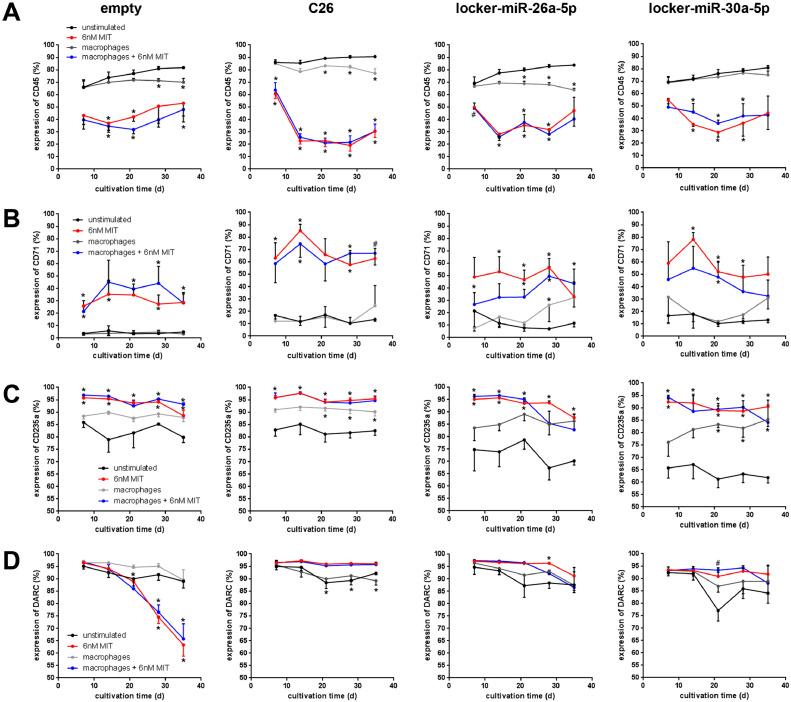
Effect of miRNA downregulation on the expression of different surface markers. **(A–D)** Fy^b-long^K562 cells were transduced with the empty locker vector, C26, locker miR-30a-5p, or locker-miR-26a-5p, and cultured under four conditions for 35 days. Every 7 days, flow cytometric analysis of CD45 **(A)**, CD71 **(B)**, CD235a **(C),** and DARC receptor **(D)** were performed. Data are presented as means ± SEM. Tests were performed two-sided. *p < 0.05 (Mann–Whitney *U* test). *N* = 4 independent experiments.

Erythroid differentiation is associated with drastic changes in the expression of different surface markers. To evaluate the impact of miR-26a-5p and/or miR-30a-5p on the expression of erythroid-specific surface markers, we performed flow cytometric analyses for CD45, CD71,and CD235a on the different cell lines after treatment with MIT in the presence or absence of macrophages. As expected, expression of the leukocyte marker CD45 is downregulated after treatment with MIT in all cell lines, with the highest effect (70% downregulation) in double-knockout cells (Figure 6A). Co-culture with macrophages also revealed, an albeit only moderate (10% downregulation), reduction in CD45 expression levels with exception in cells transduced with anti-mIR-30a-5p alone. Furthermore, downregulation of CD45 seems to be transient except in the C26 cells. In contrast, expression of CD71 und CD235a was upregulated after treatment with MIT in all cell lines, with the highest yield (70% upregulation) in double-transduced cells (Figure 6B,C). Whereas CD71 expression was not affected by the presence of macrophages, CD235a expression was significantly enhanced (Figure 6C). Interestingly, in contrast to cells transduced with anti-miR-26a-5p and/or miR-30a-5p, Duffy antigen expression was unstable in empty locker vector-transduced control cells after treatment with MIT (Figure 6D).

### Effect of mithramycin A on enucleation of double knockdown cells

**Figure 7:**
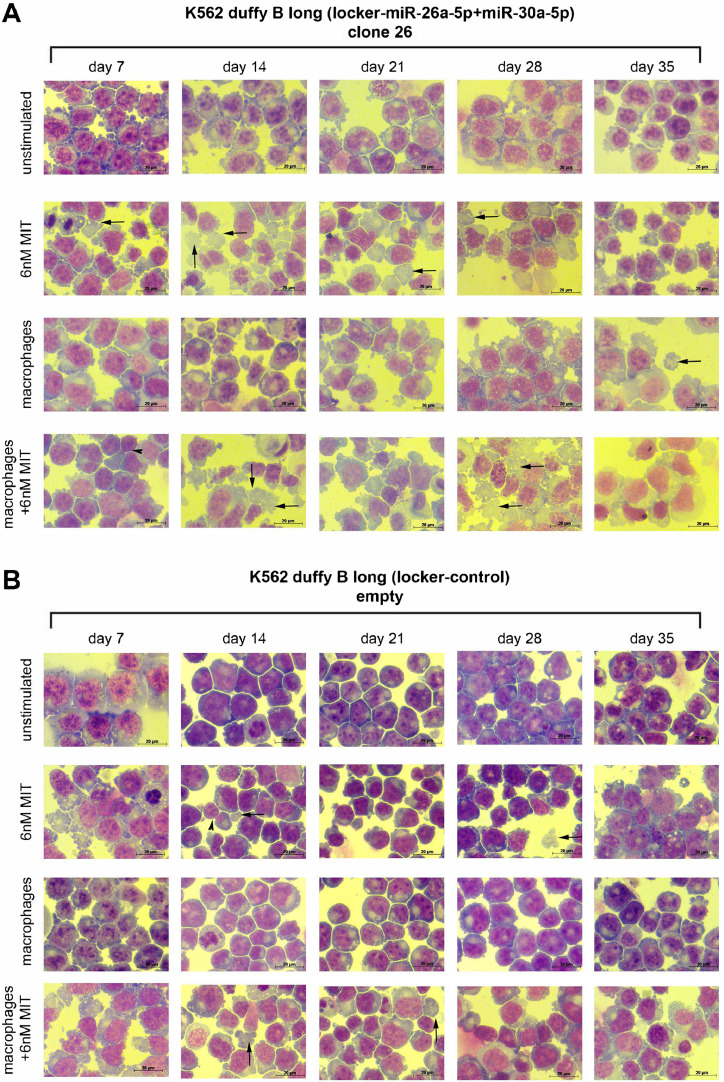
Effect of miRNA downregulation on the morphology of K562 cells during erythroid differentiation. **(A,B)** Fy^b-long^K562 cells either double transduced with locker-miR-26a-5p and locker-miR-30a-5p vectors (clone 26) **(A)** or transduced with the empty locker control vector **(B)** were cultured under four conditions for 35 days. Every 7 days, cytospins of 3 × 10^5^ cells were performed and stained with Giemsa solution. Bar = 20 µm. One of four representative experiments is illustrated.

The morphological data indicate that the treatment with Mithramycin of C26 leads to enucleation (Figure 7A, see *arrows*). The addition of macrophages, however, does not lead to an increase in the enucleation rate. The highest rate of enucleation (5%) is observed on day 21. Empty locker vector-transduced Fy^b-long^-K562 cells in which the miRNAs are not downregulated are showing enucleation as well (see *arrows*). However, a comparison of the yield in enucleated reticulocytes obtained after filtration with standard leukocyte depletion filters reveals that double-knockout Fy^b-long^-K562 cells yield 10 times the number of reticulocytes as empty locker vector-transduced Fy^b-long^-K562 cells (empty 0.5% vs. C26 5% enucleation). The typical “blebbing” is also less present in empty locker vector-transduced Fy^b-long^-K562 cells (Figure 7A,B). Macrophages alone have no influence on the enucleation process and rate. Overall, Fy^b-long^-K562-L-26a and Fy^b-long^-K562-L-30a behave in the same manner as empty locker vector-transduced cells (Supplementary Figure E4, online only, available at www.exphem.org).

### Effect of mithramycin A on expression of miRNAs in double-knockdown cells

During erythropoiesis, miR-26a-5p and miR-30a-5p are downregulated in erythroid cells [14]. MIT also induces downregulation of both miRNAs in Fy^b-long^-K562 cells (Supplementary Figure E5A, online only, available at www.exphem.org), thus contributing to enucleation. Knockdown of both miRNAs leads to downregulation by about 60%–70% as compared with the empty locker vector-transduced control (Supplementary Figure E5B, online only, available at www.exphem.org). In addition, MIT reduces expression by another 40%–60% until day 21 of cultivation. Interestingly, downregulation of only miR-30a-5p leads to a significant increase inmiR-30a-5p after treatment of cells with MIT (Supplementary Figure E5C, online only, available at www.exphem.org), whereas treatment with MIT of cells in which only miR-26a-5p was downregulated yields a moderate reduction in miR-30a-5p expression (Supplementary Figure E5D, online only, available at www.exphem.org). Expression of miR-26a-5p seems to be unaffected by treatment with MIT in both single knockdown cell lines.

## Discussion

Historically neglected because of its biological peculiarities and the absence of a continuous long-term in vitro blood stage culture system, *P. vivax* has received new attention resulting from the paradigm shift in malaria research from control to eradication.

Long-term in vitro culturing of *P. vivax* has been undermined by its inability to invade red blood cells (RBCs) other than reticulocytes, which represent as little as 0.5%–1.5% in normal blood. Procedures of enrichment from donor blood have failed to support long-term in vitro propagation, most probably because of donor variability [27], which makes it difficult for *P. vivax* to adapt. Also, the constant need for human reticulocytes from healthy donors and/or hemochromatosis patients has far-reaching ethical implications and has restricted the ability to perform short-term *P. vivax* cultures in hospital-based institutions. The continuous generation of reticulocytes from hematopoietic stem cells is also partially characterized by the same constraints of availability and possibility of long-term expansion of the same cell type to produce a stable *P. vivax*-permissive reticulocyte population over time. Furthermore, all these procedures are labor intensive, cumbersome, and low yielding.

To provide *P. vivax*-permissive reproducible reticulocytes populations, we set out to conduct studies on the differentiation and enucleation of a stable immortal cell line.

The aims of this research are thus twofold. First, it attempts to find a cell line of reticulocyte precursors allowing for simple and quick differentiation into *P. vivax*-permissive reproducible reticulocyte populations stable over time, to develop a continuous long-term blood stage culture for *P. vivax*. Second, it investigates regulatory factors behind the terminal differentiation (and enucleation, in particular) of erythroid precursors to drive the reticulocyte production. Given the twofold aim of this research, the K562 erythroleukemia cell line was chosen as a model based on some of its characteristics: it is a human, immortal cell line committed to the erythroid lineage (erythroleukemia), which is easy to culture, and has a high proliferation rate. However, it also has some major drawbacks such as the lack of surface expression of the Fy receptor needed for *P. vivax* invasion, its neoplastic character, and thus the lack of spontaneous differentiation and enucleation to produce reticulocytes.

As the invasion of *P. vivax* into reticulocytes is Fy receptor dependent [11,19], the K562 erythroleukemia cell line was engineered to stably express the different variants of the receptor on its surface. Given the extremely cumbersome access to *P. vivax*, subsequent studies to ascertain parasite binding, invasion, and growth were conducted with the *P. vivax-*type parasite *P. knowlesi* and the gold standard *P. vivax* model, *P. cynomolgi,* which is nearly identical to *P. vivax* with respect to biology [28,29]. While binding and invasion were observed, parasites do not grow past the 29 hours likely because of the lack of hemoglobin and possibly the presence of the nucleus. On the basis of the results, the Fy^b-long^-expressing K562 erytholeukemia cell line was selected for differentiation and enucleation studies. Although permissivity for these *P. vivax*-type parasites may not be definitive proof that the cells are also *P. vivax* permissive, *P. knowlesi* and *P. cynomolgi* were chosen based on the fact that they are good models for the Fy receptor-dependent invasion of red cells [20,21], where *P. cynomolgi*, in particular, is the parasite phylogenetically and biologically most closely related to *P. vivax*.

Although the various steps in the process of differentiation of hematopoietic stem cells to mature erythrocytes are relatively well documented [3,30,31], much less is known about the mechanisms underlying the process of enucleation, which is fundamental in the transition of precursors to reticulocytes.

Different miRNAs have been identified in a number of studies 32, 33, 34 as important regulatory factors in the proliferation, differentiation, and enucleation of erythroid precursors. Rouzbeh et al. [14] reported that miRNA-30a-5p is a key inhibitor of erythroid enucleation in human embryonal cell lines, while Jia et al. [35] found that the K562 erythroleukemia cell line overexpresses the known tumor marker miRNA-26a-5p, which has an important role as a regulator of differentiation in stem cells [36].

These two miRNAs were chosen as targets for downregulation in our studies aimed at the differentiation of the Fy^b-long^-expressing K562 erythroleukemia cell line into nucleus-free reticulocytes.

When compared with the empty locker vector-transduced control, the Fy^b-long^-expressing K562 erythroleukemia cell line C26 in which both miRNA-26a-5p and miRNA-30a-5p were downregulated exhibits increased signs of erythropoiesis: higher hemoglobin production, α- and γ-globin chain expression, and erythroid surface marker expression. Moreover, a tenfold increase in the enucleation rate is observed, as is stable expression of the Duffy antigen. Although this is a positive start, the number of reticulocytes produced is still rather low, making *P. vivax* invasion and growth studies cumbersome.

In the context of *P. vivax* intracellular growth, it is also important to consider that the parasite matures inside circulating reticulocytes by using hemoglobin as a source of nutrients [37]. Circulating reticulocytes are known to contain adult hemoglobin characterized by the α- and β-globin chains, while the reticulocytes obtained from the differentiation of the C26 line contain fetal hemoglobin characterized by α- and γ-globin chains. It is likely that activation of the β-globin gene may be necessary to obtain the hemoglobin more amenable to *P. vivax* digestion and thus required for sustained *P. vivax* intracellular growth.

As the role of miR-26a-5p and miR-30a-5p during erythroid differentiation was yet to be investigated, this work also set out to determine the specific influence of these miRNAs on erythropoiesis using the single-knockdown cell lines: Fy^b-long^-K562-L-26a and Fy^b-long^-K562-L-30a.

Our results confirm the tumor marker character of miRNA-26a-5p reported by Jia et al. [35]. While miRNA-26a-5p appears to have no direct influence on erythropoiesis, it is responsible for the neoplastic character of the K562 erythroleukemia cell line, where it increases the proliferation rate at the expense of differentiation. Our results suggest that the regulation of miRNA-26a-5p and miRNA-30a-5p may be intertwined as the downregulation of miRNA-26a-5p only in the Fy^b-long^-K562-L-26a cell line results in an increase in the expression of miRNA-30a-5p above the levels detected in the empty locker vector-transduced controls. Treatment of the Fy^b-long^-K562-L-26a cell line with MIT, which prompts a reduction of miRNA-30a-5p levels in empty locker vector controls, results only in an miRNA-30a-5p downregulation that is analogous to that obtained by MIT treatment in empty locker vector controls. In this context, macrophages appear to play an important role in driving the erythropoiesis as they reduce Fy^b-long^-K562-L-26a proliferation and miRNA-30a-5p expression, while increasing hemoglobin production and the erythroid morphology of the cells. In the bone marrow, reticulocytes are formed in specific niches, known as erythroblastic islands, which are composed of a central macrophage, which provides nutrients as well as proliferative and survival signals [38], surrounded by erythroblasts at different stages of maturation [1,38,39].

The differentiation of the Fy^b-long^-K562-L-26a cell lines is, however, transient as confirmed by the behavior of the surface markers, which initially exhibit a marked erythroid character that is subsequently lost. This could be explained by the development of a countermechanism by the Fy^b-long^-K562-L-26a cell line, which leads to reactivation of miRNA-26a-5p expression.

As detailed by Rouzbeh et al. [14], miRNA-30a-5p is a key regulator of enucleation. However, in Fy^b-long^-K562-L-30a, in which only miRNA-30a-5p is downregulated, only limited downregulation of the miRNA-30a-5p was observed together with a limited degree of differentiation and stable expression of miRNA-26a-5p. Particularly surprising was the increase in miRNA-30a-5p upon treatment with MIT, which was accompanied by an increase in the expression of miRNA-26a-5p. These data may be explained by the malignant character of the K562 cell line, which is maintained when miRNA-26a-5p is not downregulated and which drives the cells to proliferation rather than differentiation. To counter the erythropoietic drive deriving from downregulation of miRNA-30a-5p, the cells overexpress miRNA-30a-5p to such a degree that it compensates for the effect of anti-miRNA treatment. The cells were thus able to deploy a countermechanism to protect their neoplastic character, which also resulted in the increase in miRNA-26a-5p.

In both single-knockdown (Fy^b-long^-K562-L-26a and Fy^b-long^-K562-L-30a) and control cell lines, destabilization of the Fy antigen is observed toward the end of the experimental period (around days 28–35). An explanation for these data may also lie in the malignant character of the K562 cell line. In fact, there is evidence that the Fy antigen is a negative regulator of tumorigenesis and/or metastasis 40, 41, 42. Thus, it is likely that, in single-knockdown and control cell lines, the Fy antigen is downregulated by a countermechanism activated to protect the neoplastic character of the K562 cells.

It is clear that the data presented above suggest an intimate connection between the regulation of miRNA-26a-5p and that of miRNA-30a-5p, which makes it necessary for both miRNAs to be downregulated to achieve enucleation and stable expression of the Fy antigen in Fy^b-long^-K562 erythroleukemia cell lines. This also suggests that the regulation of erythropoiesis may be more complex than it appears. During erythropoiesis, development line-specific genes are increasingly activated, while genes of alternative development lines are inhibited. This regulation is dependent on the complex interaction between transcription factors and posttranscriptional regulators, especially miRNAs. In this context, RUNX1 is an important transcription factor at the center of the miRNA regulatory pathways needed for erythropoiesis [43]. Closely linked to its modes of action are TAL1 and GATA1, which are also considered key transcription factors in hematopoietic differentiation [44]. In addition, the transcription factors Ets1 [45] and HES1 could be identified as suppressors for erythroid development [46]. Protein arginine methyltransferase 6 (PRMT6), whose role as an inhibitor of genes necessary to erythropoiesis has only recently been revealed, is another important regulator. In this context, it has been found that the PRMT6 inhibitor MS023 promotes erythroid differentiation in K562 [47]. From this small selection of important regulatory factors, it can be shown that for many parameters of erythropoiesis, an even deeper understanding must be gained to fully elucidate the regulatory processes of erythroid differentiation. The further targeting of at least some of these factors will be necessary to increase the enucleation rate in Fy^b-long^-K562 erythroleukemia cell lines to levels compatible with long-term, continuous *P. vivax* in vitro blood stage culture and to promote the activation of β-globin genes, which are likely required for the sustained growth of *P. vivax* in vitro.

## Conclusions

Taken together, these results reveal an interplay in the mechanisms of action of miR-26a-5p and miR-30a-5p, which makes it necessary to downregulate both miRNAs to achieve a stable enucleation rate and Fy receptor expression. The tenfold increase in the enucleation rate of C26 vis-à-vis the empty vector-treated control is an interesting initial result, which can be improved upon by the targeting of other factors involved in the regulation of erythropoiesis. In the context of establishing *P. vivax*-permissive stable and reproducible reticulocytes for long-term in vitro blood stage cultures, promoting the shift in hemoglobin production from fetal to adult may also be necessary. Despite the fact that K562 erythroleukemia cell lines are of neoplastic origin, this cell line offers a versatile model system to research the regulatory mechanisms behind erythropoiesis. The complete understanding of such regulatory mechanisms would open the door to novel therapeutic approaches to malignant diseases of the erythropoietic compartment and provide insights into which regulatory mechanisms need to be targeted to obtain high numbers of viable reticulocytes for *P. vivax* culture.

## Appendix Supplementary materials

Image, application 1

## Footnotes

Supplementary material associated with this article can be found in the online version at doi:10.1016/j.exphem.2020.01.012.

## References

[1] Chasis JA, Mohandas N (2008). Erythroblastic islands: niches for erythropoiesis. Blood.

[2] Yeo JH, McAllan BM, Fraser ST (2016). Scanning electron microscopy reveals two distinct classes of erythroblastic island isolated from adult mammalian bone marrow. Microsc Microanal.

[3] Yeo JH, Lam YW, Fraser ST. Cellular dynamics of mammalian red blood cell production in the erythroblastic island niche. Biophys Rev. 10.1007/s12551-019-00579-2 PMC6874942 31418139

[4] Liu J, Guo X, Mohandas N, Chasis JA, An X (2010). Membrane remodeling during reticulocyte maturation. Blood.

[5] Liu AP, Aguet F, Danuser G, Schmid SL (2010). Local clustering of transferrin receptors promotes clathrin-coated pit initiation. J Cell Biol.

[6] Bessis M, Weed RI (1973). The structure of normal and pathologic erythrocytes. Adv Biol Med Phys.

[7] Garnham PCC (1966). Malaria parasites and other haemosporidia.

[8] Schnittger L, Rodriguez AE, Florin-Christensen M, Morrison DA (2012). Babesia: a world emerging. Infect Genet Evol.

[9] William T, Rahman HA, Jelip J (2013). Increasing incidence of *Plasmodium knowlesi* malaria following control of *P. falciparum* and *P. vivax* malaria in Sabah, Malaysia. PLoS Negl Trop Dis.

[10] Gallup JL, Sachs JD (2001). The economic burden of malaria. Am J Trop Med Hyg.

[11] Barnwell JW, Nichols ME, Rubinstein P (1989). In vitro evaluation of the role of the Duffy blood group in erythrocyte invasion by *Plasmodium vivax*. J Exp Med.

[12] Bartel DP (2004). MicroRNAs: genomics, biogenesis, mechanism, and function. Cell.

[13] Winter J, Jung S, Keller S, Gregory RI, Diederichs S (2009). Many roads to maturity: microRNA biogenesis pathways and their regulation. Nat Cell Biol.

[14] Rouzbeh S, Kobari L, Cambot M (2015). Molecular signature of erythroblast enucleation in human embryonic stem cells. Stem Cells.

[15] Ozwara H, van der Wel A, Kocken CH, Thomas AW (2003). Heterologous promoter activity in stable and transient *Plasmodium knowlesi* transgenes. Mol Biochem Parasitol.

[16] Miller LH, Hudson D, Rener J, Taylor D, Hadley TJ, Zilberstein D (1983). A monoclonal antibody to rhesus erythrocyte band 3 inhibits invasion by malaria (*Plasmodium knowlesi*) merozoites. J Clin Invest.

[17] Chitnis CE, Chaudhuri A, Horuk R, Pogo AO, Miller LH (1996). The domain on the Duffy blood group antigen for binding *Plasmodium vivax* and *P. knowlesi* malarial parasites to erythrocytes. J Exp Med.

[18] Pasini EM, van den Ierssel D, Vial HJ, Kocken CH (2013). A novel live–dead staining methodology to study malaria parasite viability. Malar J.

[19] Barnwell JW, Wertheimer SP (1989). Plasmodium vivax: merozoite antigens, the Duffy blood group, and erythrocyte invasion. Prog Clin Biol Res.

[20] Dalton JP, Hudson D, Adams JH, Miller LH (1991). Blocking of the receptor-mediated invasion of erythrocytes by *Plasmodium knowlesi* malaria with sulfated polysaccharides and glycosaminoglycans. Eur J Biochem.

[21] Kosaisavee V, Suwanarusk R, Chua ACY (2017). Strict tropism for CD71(+)/CD234(+) human reticulocytes limits the zoonotic potential of *Plasmodium cynomolgi*. Blood.

[22] Bianchi Scarra GL, Romani M, Coviello DA (1986). Terminal erythroid differentiation in the K-562 cell line by 1-beta-D-arabinofuranosylcytosine: accompaniment by c-myc messenger RNA decrease. Cancer Res.

[23] Osti F, Corradini FG, Hanau S, Matteuzzi M, Gambari R (1997). Human leukemia K562 cells: induction to erythroid differentiation by guanine, guanosine and guanine nucleotides. Haematologica.

[24] Rutherford T, Clegg JB, Higgs DR, Jones RW, Thompson J, Weatherall DJ (1981). Embryonic erythroid differentiation in the human leukemic cell line K562. Proc Natl Acad Sci USA.

[25] Salvador A, Dall'Acqua S, Sardo MS (2010). Erythroid induction of chronic myelogenous leukemia K562 cells following treatment with a photoproduct derived from the UV-A irradiation of 5-methoxypsoralen. ChemMedChem.

[26] Trakarnsanga K, Wilson MC, Lau W (2014). Induction of adult levels of beta-globin in human erythroid cells that intrinsically express embryonic or fetal globin by transduction with KLF1 and BCL11A-XL. Haematologica.

[27] Sandberg S, Rustad P, Johannesen B, Stolsnes B (1998). Within-subject biological variation of reticulocytes and reticulocyte-derived parameters. Eur J Haematol.

[28] Joyner C, Barnwell JW, Galinski MR (2015). No more monkeying around: primate malaria model systems are key to understanding *Plasmodium vivax* liver-stage biology, hypnozoites, and relapses. Front Microbiol.

[29] Schmidt LH (1983). Appraisals of compounds of diverse chemical classes for capacities to cure infections with sporozoites of *Plasmodium cynomolgi*. Am J Trop Med Hyg.

[30] Andersson LC, Jokinen M, Gahmberg CG (1979). Induction of erythroid differentiation in the human leukaemia cell line K562. Nature.

[31] Silva G, Cardoso BA, Belo H, Almeida AM (2013). Vorinostat induces apoptosis and differentiation in myeloid malignancies: genetic and molecular mechanisms. PloS One.

[32] Felli N, Fontana L, Pelosi E (2005). MicroRNAs 221 and 222 inhibit normal erythropoiesis and erythroleukemic cell growth via kit receptor down-modulation. Proc Natl Acad Sci USA.

[33] Wang Q, Huang Z, Xue H (2008). MicroRNA miR-24 inhibits erythropoiesis by targeting activin type I receptor ALK4. Blood.

[34] Yang GH, Wang F, Yu J, Wang XS, Yuan JY, Zhang JW (2009). MicroRNAs are involved in erythroid differentiation control. J Cell Biochem.

[35] Jia LF, Wei SB, Gan YH (2013). Expression, regulation and roles of miR-26a and MEG3 in tongue squamous cell carcinoma. Int J Cancer.

[36] Su X, Liao L, Shuai Y (2015). MiR-26a functions oppositely in osteogenic differentiation of BMSCs and ADSCs depending on distinct activation and roles of Wnt and BMP signaling pathway. Cell Death Dis.

[37] Rawat M, Vijay S, Gupta Y, Dixit R, Tiwari PK, Sharma A (2011). Sequence homology and structural analysis of plasmepsin 4 isolated from Indian *Plasmodium vivax* isolates. Infect Genet Evol.

[38] Manwani D, Bieker JJ (2008). The erythroblastic island. Curr Top Dev Biol.

[39] Bessis M (1958). [Erythroblastic island, functional unity of bone marrow]. Rev Hematol.

[40] Maeda S, Kuboki S, Nojima H (2017). Duffy antigen receptor for chemokines (DARC) expressing in cancer cells inhibits tumor progression by suppressing CXCR2 signaling in human pancreatic ductal adenocarcinoma. Cytokine.

[41] Massara M, Bonavita O, Mantovani A, Locati M, Bonecchi R (2016). Atypical chemokine receptors in cancer: friends or foes?. J Leukoc Biol.

[42] Wang J, Ou ZL, Hou YF (2006). Enhanced expression of Duffy antigen receptor for chemokines by breast cancer cells attenuates growth and metastasis potential. Oncogene.

[43] Rossetti S, Sacchi N (2013). RUNX1: A microRNA hub in normal and malignant hematopoiesis. Int J Mol Sci.

[44] Kuvardina ON, Herkt S, Meyer A (2017). Hematopoietic transcription factors and differential cofactor binding regulate PRKACB isoform expression. Oncotarget.

[45] Sieweke MH, Tekotte H, Frampton J, Graf T (1996). MafB is an interaction partner and repressor of Ets-1 that inhibits erythroid differentiation. Cell.

[46] Ishiko E, Matsumura I, Ezoe S (2005). Notch signals inhibit the development of erythroid/megakaryocytic cells by suppressing GATA-1 activity through the induction of HES1. J Biol Chem.

[47] Herkt SC, Kuvardina ON, Herglotz J (2018). Protein arginine methyltransferase 6 controls erythroid gene expression and differentiation of human CD34(+) progenitor cells. Haematologica.

