# Supplementary material for: K562 erythroleukemia line as a possible reticulocyte source to culture Plasmodium vivax and its surrogates
Source: Exp Hematol. 2020 Feb;82:8–23. doi: 10.1016/j.exphem.2020.01.012 (PMC7097847; doi:10.1016/j.exphem.2020.01.012)
Supplement: Supplementary file 1 [file mmc1.docx]

**Materials and methods supplementary materials**

**Chemicals and antibodies**

Holo-transferrin was purchased from R&D (Minneapolis, USA). Hydrocortison, insulin (solution) and blasticidine S were purchased from Sigma/Aldrich (Schnelldorf, Germany). Mithramycin A was purchased from Biomol GmbH (Hamburg, Germany). Recombinant human erythropoietin (EPO) was purchased from PeproTech (Hamburg, Germany). Mithramycin (Brunschwig Chemie, The Netherlands); Aphidicolin (Santa Cruz, USA), Valproic acid (Sigma); Resveratrol (Sigma); Hydroxyurea (Sigma); Nicotinamide (Sigma); 5-Azacytidine (Sigma); Antimony(III)oxide (Sigma); Vinblastinsulfate (Santa Cruz, USA); Chromomycin A (Calbiochem); Butyric acid (Sigma); Vanadium(V)oxide (Sigma); Aclacinomycin A (Santa Cruz, USA), Idarubicin hydrochloride (Santa Cruz, USA), Daunorubicin hydrochloride (Santa Cruz, USA), Pirarubicin (Santa Cruz, USA), Aloin (Santa Cruz, USA). In the FACS analysis of the chemically-induced K562 differentiation the following antibodies were used: DARC-APC (R&D Systems, Clone # 358307); CD45-V450 (Becton Dickinson, Clone # HI30); CD71-PE (Becton Dickinson, Clone # M-A712); CD235a-APC **(**Becton Dickinson, GA-R2).

**Cultivation of cell lines, plasmids and production of viral supernatants.**

Human embryonic kidney 293T cells (DSMZ, Braunschweig, Germany) were cultured in Dulbecco's modified Eagle's medium with stable glutamine and high glucose (Life technologies, Darmstadt, Germany) supplemented with 10% fetal calf serum (FCS), 100 U/ml penicillin, 100 μg/ml streptomycin. K562 cells were acquired from DSMZ and tested negative for mycoplasma contamination. K562 cells were cultured in complete RPMI-1640 consisting of RPMI-1640 (Biochrom) supplemented with 10% FCS, 1% L-glutamine, 100 U/ml penicillin, 100 μg/ml streptomycin, 1% sodium pyruvate (Biochrom) and 1% NEM-non-essential amino acids (Biochrom).

Viral supernatants were produced by transient transfection of 293T cells. Briefly, 293Tcells were incubated with 21 μg of pLV-[mir-locker] (Biosettia, San Diego, USA) or SEW-Fya-L-P2A-Fy-variant 7 μg of the vesicular stomatitis virus envelope glycoprotein envelope plasmid (pMD2.G) and 14 μg of the packaging plasmid pR8.94 for 12-16h. The following day, the medium was replaced with fresh medium. Supernatants were collected 48h after transfection, filtered through 0.45 μm filters (Corning, NY, USA), concentrated with PEG-it virus precipitation solution, and stored at -80 °C until further usage. Viral stocks were titrated by transduction of K562 cells for 48h using different dilutions of the virus. Flow cytometric analysis was used to determine the percentage of GFP-fluorescent cells obtained using the different dilutions of the viral stocks.

**Generation of different Duffy variant constructs.**

The primers list for the generation of Duffy variants is provided in supplementary table 1. First a mini gene including a synthetic intron (pCI-Intron) and the codon optimized cDNA for the long Duffy transcript variant for allele B (Fy-BL) was synthetized by Geneart (Life technologies). A short Duffy variant (Fy-Bs) comprising modifications on the sequence at residues 1-9 of the amino terminal end of the long Duffy variant (Fy-BL) was engineered by two sequential mutagenesis PCR steps using the QuikChange II Site-Directed Mutagenesis Kit (Agilent) and the plasmid pMA-T-Fy-BL (GeneArt®, Life technologies) as template with the primer set Duffy 2-7. The resulting intermediate plasmid pMA-T-Fy-BL-mod_2-7 was used in the next mutagenesis PCR as template to delete the residues at position 8 and 9 with the primer set del_8-9 resulting in generation of pMA-T-Fy-Bs. Finally, the introduction of an amino acid substitution from Asp to Gly at position 44 into the short and long Duffy (FyB) variants led to the generation of Fy-As and Fy-AL enclosing allele A by using the mutagenesis primer set D44G on the short and long Duffy variants. After generation of Duffy variants, the coding sequence for Duffy including the synthetic intron was excised from the parental plasmid by digestion with BamHI and SbfI and inserted in the BamHI and SbfI restriction sites of the lentiviral vector SEW resulting in lentiviral vectors coding for the different Duffy variants. For the generation of the SEW-Fy-AL-p2A-Fy-BL first, the stop codon in the Fy-AL ORF was deleted by PCR with the primers BamHI-pCI-F and SbfI-Fy(dUGA)-R and subsequent ligation of the BamHI/SbfI digested PCR product with the BamHI/SbfI sites in the pSEW vector, resulting in pSEW-Fy-AL(dUGA). Then the P2A self-cleaving peptide sequence was inserted as a 5´overhang in the forward primer that is targeting the amino terminal end of Fy-AL. The corresponding long oligonucleotide referred as SbfI-(**c**)-2A-FyB-F was used with Fy-BL template and the reverse primer SbfI-Fy-R to attach P2A to Fy-BL in the cloning PCR. The resulting PCR product was digested with SbfI and ligated with the SbfI linearized intermediate pSEW-Fy-AL(dUGA) vector. Finally, clones with right orientation of SbfI-c-P2A-FyB-F-TGA-SbfI downstream to Fy-AL(dUGA) resulting in pSEW-SFFV-Fy-AL(dUGA)-SbfI-c-P2A-Fy-BL(UGA)-SbfI cassette organization were screened by restriction analysis and confirmed by sequencing analysis, additionally.

**Lentiviral transduction and selection of single cell clones**

The day before transduction, K562 or *Fy*-K562 cells were plated at a density of 2 x 10^5^/well in a 12 well plate. For transduction, K562 or *Fy*-K562 cells were spin-infected for 30 minutes at 800 *g* and 32°C with lentiviral supernatants at an MOI of 10-20, and then cultured for 48 hours in the absence of antibiotics. In case of transduction with pLV-[locker-miRNA] cells were then selected with 25µg/mL blasticidine S

**Selection of single cell clones**

For the selection of single cell clones of Duffy variant expressing K562 cells and later miRNA double knock-downs transduced *Fy*-K562 cells were seeded (250 cells per well) in Methocult®H4100 (Stem Cell Technologies inc., Vancouver, BC, Canada) supplemented with complete RPMI-1640 medium. Single cell colonies were imaged and counted with a STEMvision™ instrument (Stem Cell Technologies Inc.). Single cell clones were picked, expanded in complete RPMI-1640 culture medium and treated with 1.5µM SAHA. Only clones which expressed high HbF levels were used for miRNA double knockdown. For the analysis of miRNA expression levels, the selection of single cell clones was performed as described above. Clones found to express the lowest levels of miR-26a-5p and miR-30a-5p were used in further investigations.

**Isolation of *P. knowlesi* parasites**

**Isolation of *P. knowlesi* and *P. cynomolgi* parasites**

*In vitro* wild type or GFP-labelled (1) *P. knowlesi* parasites *H* strain cultures (2) were synchronized using two cycles of 0,3M alanine treatment (3) and enriched to high parasitemia (7-10%) by lowering the hematocrit to about 2.5%. The medium was changed once or twice a day depending on the parasite’s growth phase to keep the culture healthy. *P. knowlesi* schizonts were purified using either the MACS as described previously (4) or an adaptation of the methodology described by Salmon et al. (5), which also yielded merozoites for subsequent studies.

Briefly, schizont stage parasite cultures were transferred into a 50 mL conical tube, washed twice in RPMI and resuspended in 9X pellet volume with RPMI. 5 mL of resuspended culture were carefully layered onto a 2.5 mL 40-70% Percoll (GE Healthcare) cushion in a 15 mL tube and centrifuged at 1000 x g on a swing out rotor Allegra X-15R centrifuge for 16 minutes without brake. Thereafter, the layer between the 40% and 70% gradient (brownish layer) was carefully aspirated into a clean tube and washed three times with ice-cold RPMI at 1000 x g for 5 min (medium brake) at 4 °C each time to remove traces of Percoll. The harvested gradient was resuspended in 10X volume of culture medium containing E64 (1:1000, Sigma) and put back in culture for 2-6 hours in the incubator for all the schizonts to maximally ripen into merozoites. Thereafter, the culture was harvested and washed three times with ice-cold RPMI at 1000 x g for 5 minutes each time to remove traces of E64 and harvest intact, mature schizonts. The supernatant was then centrifuged at high speed (10 min at 1,900 × *g*) to pellet the merozoites. The purity and number of merozoites per schizonts was assessed by a thin blood smear and merozoites were counted using a Bruker chamber

**Isolation of *P. cynomolgi* parasites**

For the isolation of *P. cynomolgi* parasites, brown layers derived from an in vivo macaque infection with wild-type (M strain) or transgenic fluorescent *P. cynomolgi* (6) were synchronized using the MACS technology according to Trang DT et al. (7). The tightly synchronized, very mature schizonts were resuspended in a buffer mimicking intracellular ionic conditions and merozoites were harvested as described by Bansal et al. (8).

Briefly, schizonts were allowed to rupture and release merozoites. Cultures containing unruptured schizonts and released merozoites were centrifuged at 500 × g in a Beckman Coulter centrifuge for 5 min to separate released merozoites from unruptured schizonts and uninfected erythrocytes. Supernatant containing free merozoites was centrifuged at 3300 × g using an Eppendorf 5427R centrifuge for 5 min to collect merozoites. The merozoites were resuspended in RPMI 1640 medium (incomplete RPMI) or IC buffer for use in experiments.

**Duffy binding and invasion studies with Plasmodium spp.**

Both wild type and transgenic fluorescent parasites (1) isolated as described were used for binding and co-culture assays. In this set of experiments the K562 mother line not transduced with Duffy was taken along as a control.

Binding assays were carried out either in suspension or using K562 monolayers.

Assays to assess *P. knowlesi* and *P. cynomolgi* merozoite binding to different variants of Fy-K562 in suspension were performed using a methodology adapted from Miller et al. (9) and Chitnis et al. (10).

Briefly, mature *P. knowlesi* or *P. cynomolgi* schizont-infected erythrocytes (10^7^/mL) purified using MACS technology as previously described (4) were added to a 50 µL suspension of the specific Fy-K562 variant (10^8^/mL) to be tested in 96-well flat bottom plates (2 sets of quadruplicates per *Fy* variant). The plates were shaken in an incubator (New Brunswick Scientific, Innova 44) at 37C for between 1 and 3hrs to mix the suspensions well and allow the merozoites to egress and adhere. Small samples were taken between 1h and 3hrs at 15 minutes intervals each hour, transferred to an Eppendorf tube and spun down. Non-adherent merozoites were removed by resuspending the pellet in PBS three times, followed by pelleting each time. Giemsa stained thin smears were made to assess parasite binding using an UV-microscope at each timepoint.

Alternatively, purified merozoites (2,5x10^8^) were stained with 1 µL Hoechst-dye in Ringers and incubated 30 minutes in an Eppendorf tube or a 96-well flat bottom plate with the specific Fy-K562 (10^8^ mL) variant to be tested. Thereafter, the mix was spun down and washed three times in PBS at 1,500 rpm for 1 min each time. Thin smears on glass slides were made from Eppendorf samples and stained with Giemsa to assess binding under the UV-microscope. 96-well flat bottom plates were assessed using an operetta according to a methodology described before in Pasini et al. (11). The experiment was repeated 9 times per variant for each parasite species; Fy-variants were plated in quadruplicates.

**Duffy binding assays with fixed K562 monolayers**

Plates coated with three different materials corresponding to cationic (poly-L-Lysine (Sigma-Aldrich P6407)), electrostatic (PVC (Brunchwig Chemie 34-7717205)) and anionic binding (Aspartame (Sigma-Aldrich PHR1381)) respectively were used to define the optimal binding conditions for *Fy*-transduced K562 cell lines. Plates were coated as follows: half a 96-well flat bottom plate was coated with 20 µL/well poly-L-Lysine (concertation range: 0,05-0,8 mg/mL; two-fold dilutions steps) or aspartame (concentration rage: 0,1- 1 mg/mL) by incubating the plate for approximately 2 hours on a rocking platform at room temperature. Thereafter, excess fluid was removed, plates were washed with cold PBS pH 7.4 and left to dry in a flow hood overnight. The other half plate was left uncoated. PVC coated plates were purchased commercially.

Fy-transduced K562 cells suspended in 250 µL complete medium were seeded across coated and non-coated wells in 96-well flat bottom plates prepared as described above in a range of 1,6 10^4 to 2,04 10^6 cells per well using two-fold dilution steps and tested for their ability to form discrete monolayers. The cells were allowed to settle for 2h, rested for additional 30 minutes and then observed under an inverted microscope. 2 x 10^5 K562 cells/ per well was chosen as the ideal density for seeding the cells to obtain reproducible monolayers in 96-well flat bottom plates.

For the fixation of K562 monolayers 2 x 10^5 K562 cells/ per well were seeded in an aspartame coated 96-well flat bottom plate as described above and fixed by either cautiously removing the medium and adding 100 µL 100% methanol/ethanol or 2% para-formaldehyde/formaldehyde in PBS pH 7.4; alternatively, only 100µL complete medium were removed and 100 µL 4% para-formaldehyde or formaldehyde in PBS pH 7.4 were added. The plates were incubated for 35 minutes at room temperature, after which the fixative was removed and they were vigorously washed before checking for the stability of the monolayer under an inverted microscope. If the monolayer was found to be integer, plates were blocked overnight with 1% BSA/PBS at 4°C. The next morning plates were washed 3 times in PBS to remove traces of block buffer and used for ELISA aimed at checking the integrity of the DARC receptor after fixation or for binding assays.

To check integrity of Fy-receptor after fixation ELISA was performed. The anti-DARC antibody (Abcam ab58965) was diluted to 5 µg/mL in dilution buffer (PBS pH 7.4, 0,05% Tween-20, 0,5% BSA) and 1:5 serial dilutions were made in a 96-well flat bottom plate prepared above. Wells in which the anti-DARC antibody was not added were taken along as controls. The plates were incubated for 1h on a shaking platform at 450 rpm at room temperature. Thereafter, they were washed 5 times with PBS and 100 µl/well of 1∶1250-diluted goat anti-rabbit IgG/alkaline phosphatase conjugate (Pierce, Rockford, IL) was added for 1 h. Plates were then washed, incubated with 100 µl/well p-nitrophenyl phosphate (pNPP; Fluka, Poole, UK) as substrate for 30 min. and the optical density (OD) at 405 nm read with a 96-well ELISA plate reader (BioRad, Japan).

Assays using K562 monolayers were performed after the ideal plate coating, seeding density and fixing conditions were determined. The integrity of Fy-receptor after fixation was checked by ELISA using an anti-DARC antibody (Abcam ab58965). In our hands: 5 x 10^5 Fy-K562 cells/ per well on a plate coated with 0,8 mg/mL aspartame fixed with 4% para-formaldehyde gave the best Fy-K562 monolayers, while a ratio of 2.5x isolated merozoites per K562 cell gave the clearest binding study results.

Plates were incubated at room temperature on a shaking platform for 30 minutes and then allowed to settled for 1h without shaking. The supernatant was removed from the wells and the cells were washed 3x with PBS. Plates incubated with WT merozoites were stained with stained with Giemsa and counted under the normal microscope. Plates incubated with transgenic fluorescent parasites were further incubated with 1 µL/well Hoechst-dye in Ringers for 30 minutes at room temperature. Thereafter, the 96-well flat bottom plates were assessed using an operetta according to a methodology described before in Pasini et al. (11). The experiment was repeated 6 times per variant for each parasite species; Fy-variants were plated in quadruplicates.

**Co-culture of *P. cynomolgi/P. knowlesi* and Duffy B long-positive K562 cells**

Briefly, mature *P. knowlesi* or *P. cynomolgi* schizont-infected erythrocytes (10^7^/mL) purified using MACS technology as previously described (4) were added to a 50 µL suspension of the specific Fy-K562 variant (10^8^/mL) to be tested in 96-well plates (2 sets of quadruplicates) and shaken in an incubator (New Brunswick Scientific, Innova 44) at 37C. Small samples were taken at timepoints 15 minutes, 20 minutes, 30 minutes, 8h and 29h, washed once with 100% fetal calf serum and pelleted. Giemsa stained thin smears were made to assess parasite invasion and development at each timepoint.

**Induction of the erythroid differentiation using chemicals**

In preliminary differentiation experiments, cells were stimulated with RPMI-based K562 medium supplemented with 10% human AB serum, 1% Penicillin/Streptomycin, 1% L-Glutamine, 1% MEM Non-Essential Amino Acids Solution, 1% Sodium Pyruvate and different combinations of the following substances in the presence or absence of SAHA (1,5µM) and Ara-C (100nM) (Table 1), respectively: 330 μg/ml of holo-Transferrin, 3 U/ml erythropoietin, 10 μg/ml Insulin and 5ng/ml IL-3. Subsequently, an additional twelve chemicals, chosen based on a literature review of all chemicals ever successfully used in the differentiation of the erythroleukemia K562 cell line or other erythroleukemia cell lines (e.g. KU-812(F)) (12), were tested at concentrations indicated in Table 1 (13, 14 , 15) for their potential to induce differentiation and enucleation of Fy-K562. The chemically untreated Fy-K562 line was used as a control. In comparison to the preliminary experiments described above, 1% Penicillin/Streptomycin was replaced by 15 µg/ml gentamycin and the medium was not supplemented with IL-3. 1µM hydrocortison was added to the medium during the first 14 days of differentiation. The grade of erythroid differentiation was monitored by morphological analysis, expression of surface marker (CD45, CD71, DARC, CD235a) and hemoglobin production. A FACS microtiter plate technology was developed, which allowed the monitoring of differentiation over a 21-day period using FACS-based cell surface marker measurements and benzidine staining. The HbF levels in Fy-K562 cells displaying a high Hb production by benzidine staining were further confirmed by FACS analysis using the FMH Quick Quant kit according to the manufacturer´s instructions. The selection was made to continue with chemicals able to promote a high Hb production, the down regulation of CD45 (a lymphoid marker), the upregulation of the erythroid markers CD71 and CD325a, while leaving DARC unchanged.

**Details of microtiter methodology developed for testing the influence of chemicals on Fy-K562 differentiation**

In order to test the ability of these chemicals to promote differentiation their effect on specific surface markers (CD45, CD71, DARC, CD235a) was monitored using a purpose developed FACS microtiter plate technology. Briefly, Fy-K562 were seeded at a concentration of 5x10^4 cells/well in 96-wells U-bottom plates (200µL total well volume) in triplicate and chemicals were added at a chosen concentration over a 21-day period for the differentiation experiment. Measurements of the 4 different surface cell markers and benzidine stainings were performed at 2- to 4-day intervals following the schedule: t=0, t=3, t=7, t=9, t=11, t=14, t=16, t=18, t=21. These data were evaluated in combination with the results of the benzidine staining test performed as described below, which is commonly used to establish the level of hemoglobin production. For quick monitoring of promising chemicals and concentrations to be used in follow-up experiments, the amount of Hb produced in chemically treated Fy-K562 in comparison to the untreated Fy-K562 were evaluated by counting benzidin positive cells. The HbF levels in Fy-K562 cells displaying a high Hb production were further confirmed by FACS analysis using the FMH Quick Quant kit according to the manufacturer´s instructions.

**Induction of the erythroid differentiation in Fy-K562 microRNA downregulated clones**

Selected chemicals (Aphidicolin, Ara-C, Mithramycin A and SAHA) were tested for their effect in the induction of the differentiation of microRNA downregulated Fy-K562 clones and the best (Mithramycin A) was chosen for testing differentiation in the presence of macrophages. The Fy-K562 in which microRNA were downregulated resulted more sensitive to Mithramycin A than the original Fy-K562: an intermediate concentration of 6nM was therefore used.

For induction of the erythroid differentiation Fy-K562 in which microRNA were downregulated were grown in complete RPMI-1640 medium described above, supplemented with 330 μg/mL of holo-transferrin, 3 U/mL EPO and 10 μg/mL insulin. FBS was substituted by 10% human AB Serum (DRK blood donation service center Ba-Wü,Ulm). 1µM hydrocortison was added to the medium during the first 14 days of differentiation. Differentiation was carried out for 35 days in parallel both with and without macrophages in coculture, as well as with and without treatment with 6nM mithramycin A and differentiation stage was evaluated every 7 days. Cell counts were adjusted to 5 × 10^4^ and 1 x 10^5^ cells/mL on day 0 and 7, respectively. From day 14 cell counts were adjusted every 7 days to 2.5 x 10^5^ cells/mL. Every four days cell cultures were diluted 1:2 with fresh differentiation medium.

**RNA isolation and quantitative reverse transcription polymerase chain reaction (qRT-PCR)**

Total RNA was extracted from 5 x 10^6^ cells using a Pax Gene Blood miRNA kit (PreAnalytiX, Hombrechtikon, Swizerland). Reverse transcription of mRNA was performed using a Revert Aid H Minus First Strand cDNA Synthesis Kit according to the manufacturer´s instructions (Fisher Scientific, Schwerte, Germany). For qPCR the primers and probe are listed in Supplementary Table 2. GAPDH expression was used as the endogenous control.

For measurement of miRNA expression, reverse transcription was performed with TaqMan MicroRNA Reverse Transcription Kit using Megaplex primer pool v3.0 and, subsequently, specific TaqMan MicroRNA Assays for miRNA-30a-5p and -26a-5p (Applied Biosystems). Realtime PCR was performed using the TaqMAN Universal PCR Master Mix according to manufacturer’s specifications. For miRNAs, U6 snRNA was used as the endogenous control. Gene expression was analyzed using ΔΔCT method and normalized to untreated control.

**Monocyte isolation and differentiation into macrophages**

Monocytes, obtained from buffy coats of healthy donors were first isolated by centrifugation over Biocoll and subsequently selected using LS-MACS columns (Miltenyi Biotech) filled with CD14^+^-binding magnetic microbeads (purity: 94% ± 3%). In order to differentiate the monocytes obtained into mature macrophages, cells were plated at a density of 1.2 x 10^5^ cells/cm^2^ and cultured for 7 days in RPMI-1640 medium supplemented with 10 % human AB serum, 50µM 2-mercaptoethanol, 1% L-glutamine, 0.4 % MEM vitamine, 100 U/ml penicillin and 100 μg/ml streptomycin.

**Morphological analysis and benzidine staining**

For morphological analysis 2.5 x 10^5^ cells were suspended in PBS with Ca^2+^ and Mg^2+^ and spun down at 55 *g* without break on polysine coated glass slides. Slides were dried, fixed with methanol and stained with 20 % Giemsa staining solution.

For benzidine staining of hemoglobin bearing cells, 5 x 10^5^ cells were suspended in equal volumes of PBS with Ca^2+^ and Mg^2+^ and 13.3% benzidine staining solution (0.4% benzidine in 12% glacial acetic acid) and 6.7% perdogen and spun down at 55 *g* without break on superfrost glass slides.

Morphology and hemoglobin positive cells were analyzed with a Zeiss Primo Vert Inverted Microscope.

**Flow cytometry**

Cells were co-stained with PE-conjugated anti-CD71, APC-conjugated anti-CD235a and V450-conjugated anti-CD45, all from BD Bioscience, Heidelberg, Germany. Stable expression of the Duffy antigen (F_y_) was assessed with APC-conjugated anti-DARC (Fy) antibody in a separate sample (R&D Systems). Transduced cells were identified by GFP, which is present by default into the pLV-[mir-locker] vectors. Analyses were performed on a FACSCanto II and/or LSR II flow cytometer (BD Bioscience) with FlowJo Software. To determine the HbF, a FMH Quick Quant kit was used according to manufacturer’s specifications.

**Ethics statement**

**Study approval and Ethics**

Donor materials were provided by the German Red Cross Donation Service North-East, Institute for Transfusion Medicine (Dresden) and were used in accordance with the guidelines approved by the Ethics Committee of the Technical University of Dresden. The informed consent was obtained from all donors. Parasite materials were sourced from infections in non-human primates for parasites that cannot be cultured in vitro (*P. cynomolgi* M strain) and from in vitro cultures (*P. falciparum* NF54, *P. knowlesi* H strain). To make the best use of the parasite material derived from non-human primate infections, *P. cynomolgi* experiments were timed in such a way as to share parasite material with other projects involving *P. cynomolgi*. All non-human primate infections were carried out in accordance with European and Dutch law after a positive advice from the ethical committee (DEC). The BPRC is AAALAC accredited.

Non-human primates were used because no other models (in vitro or in vivo) were suitable for the aims of this project. The local independent ethical committee constituted conform Dutch law (BPRC Dier Experimenten Commissie, DEC) approved the research protocol (agreement number DEC# 708) prior to the start and the experiments were all performed according to Dutch and European laws. The Council of the Association for Assessment and Accreditation of Laboratory Animal Care (AAALAC International) has awarded BPRC full accreditation. Thus, BPRC is fully compliant with the international demands on animal studies and welfare as set forth by the European Council Directive 2010/63/EU, and Convention ETS 123, including the revised Appendix A as well as the ‘Standard for humane care and use of Laboratory Animals by Foreign institutions’ identification number A5539-01, provided by the Department of Health and Human Services of the United States of America’s National Institutes of Health (NIH) and Dutch implementing legislation. The rhesus monkeys (*Macaca mulatta*, either gender, age 4–7 years, Indian or mixed origin) used in this study were captive-bred and socially housed. Animal housing was according to international guidelines for nonhuman primate care and use. Besides their standard feeding regime, and drinking water ad libitum via an automatic watering system, the animals followed an environmental enrichment program in which, next to permanent and rotating non-food enrichment, an item of food-enrichment was offered to the macaques daily. All animals were monitored daily for health and discomfort. All intravenous injections and large blood collections were performed under ketamine sedation, and all efforts were made to minimize suffering.

**References**

1. Ozwara H, van der Wel A, Kocken CH, Thomas AW. Heterologous promoter activity in stable and transient Plasmodium knowlesi transgenes. Molecular and biochemical parasitology. 2003 Aug 11;130(1):61-4.

2. Kocken CH, Ozwara H, van der Wel A, Beetsma AL, Mwenda JM, Thomas AW. Plasmodium knowlesi provides a rapid in vitro and in vivo transfection system that enables double-crossover gene knockout studies. Infection and immunity. 2002 Feb;70(2):655-60.

3. Naughton JA, Bell A. Studies on cell-cycle synchronization in the asexual erythrocytic stages of Plasmodium falciparum. Parasitology. 2007 Mar;134(Pt 3):331-7.

4. Boyle MJ, Wilson DW, Richards JS, Riglar DT, Tetteh KK, Conway DJ, et al. Isolation of viable Plasmodium falciparum merozoites to define erythrocyte invasion events and advance vaccine and drug development. Proc Natl Acad Sci U S A. 2010 Aug 10;107(32):14378-83.

5. Salmon BL, Oksman A, Goldberg DE. Malaria parasite exit from the host erythrocyte: a two-step process requiring extraerythrocytic proteolysis. Proc Natl Acad Sci U S A. 2001 Jan 2;98(1):271-6.

6. Voorberg-van der Wel A, Zeeman AM, van Amsterdam SM, van den Berg A, Klooster EJ, Iwanaga S, et al. Transgenic fluorescent Plasmodium cynomolgi liver stages enable live imaging and purification of Malaria hypnozoite-forms. PloS one. 2013;8(1):e54888.

7. Trang DT, Huy NT, Kariu T, Tajima K, Kamei K. One-step concentration of malarial parasite-infected red blood cells and removal of contaminating white blood cells. Malar J. 2004 Mar 17;3:7.

8. Bansal A, Singh S, More KR, Hans D, Nangalia K, Yogavel M, et al. Characterization of Plasmodium falciparum calcium-dependent protein kinase 1 (PfCDPK1) and its role in microneme secretion during erythrocyte invasion. J Biol Chem. 2013 Jan 18;288(3):1590-602.

9. Miller LH, Hudson D, Rener J, Taylor D, Hadley TJ, Zilberstein D. A monoclonal antibody to rhesus erythrocyte band 3 inhibits invasion by malaria (Plasmodium knowlesi) merozoites. J Clin Invest. 1983 Oct;72(4):1357-64.

10. Chitnis CE, Chaudhuri A, Horuk R, Pogo AO, Miller LH. The domain on the Duffy blood group antigen for binding Plasmodium vivax and P. knowlesi malarial parasites to erythrocytes. The Journal of experimental medicine. 1996 Oct 1;184(4):1531-6.

11. Pasini EM, van den Ierssel D, Vial HJ, Kocken CH. A novel live-dead staining methodology to study malaria parasite viability. Malar J. 2013 Jun 7;12:190.

12. Nakazawa M, Mitjavila MT, Debili N, Casadevall N, Mayeux P, Rouyer-Fessard P, et al. KU 812: a pluripotent human cell line with spontaneous erythroid terminal maturation. Blood. 1989 May 15;73(7):2003-13.

13. Bianchi Scarra GL, Romani M, Coviello DA, Garre C, Ravazzolo R, Vidali G, et al. Terminal erythroid differentiation in the K-562 cell line by 1-beta-D-arabinofuranosylcytosine: accompaniment by c-myc messenger RNA decrease. Cancer Res. 1986 Dec;46(12 Pt 1):6327-32.

14. Luisi-DeLuca C, Mitchell T, Spriggs D, Kufe DW. Induction of terminal differentiation in human K562 erythroleukemia cells by arabinofuranosylcytosine. J Clin Invest. 1984 Sep;74(3):821-7.

15. Cioe L, McNab A, Hubbell HR, Meo P, Curtis P, Rovera G. Differential expression of the globin genes in human leukemia K562(S) cells induced to differentiate by hemin or butyric acid. Cancer Res. 1981 Jan;41(1):237-43.

**Supplementary Table 1.** Primers list used for the generation of different Duffy variants

| **Gene** | **Primer direction** | **Primer sequence 5‘ → 3‘** |
| --- | --- | --- |
| Duffy 2-7 | forward | CTT TCT CTC CAC AGG CCA CCA TGG GCA ACT GCC TAC ATC GGC TGC AGG CCG AGC TGA GCC CCA GC |
|  | reverse | GCT GGG GCT CAG CTC GGC CTG CAG CCG ATG TAG GCA GTT GCC CAT GGT GGC CTG TGG AGA GAA AG |
| del_8-9 | forward | CTG CCT ACA TCG GGC CGA GCT GAG CC |
|  | reverse | GGC TCA GCT CGG CCC GAT GTA GGC AG |
| D44G | forward | CGA CGG CGA CTA CGG CGC CAA TCT GGA AG |
|  | reverse | CTT CCA GAT TGG CGC CGT AGT CGC CGT CG |
| BamHI-pCI-F | forward | CCC CCC CCC GGA TCC GTA AGT ATC AAG GTT ACA AG |
| SbfI-Fy(dUGA)-R | reverse | GGG GGG GGG CCT GCA GGG CTC TTG GAG CCC AGG GTG TC |
| SbfI-(**c**)-2A-FyB-F | forward | GGG GGG GGG CCT GCA GG**C** GGA AGC GGA GCT ACT AAC TTC AGC CTG CTG AAG CAG GCT GGA GAC GTG GAG GAG AAC CCT GGA CCT ATG GCC AGC AGC GGC TAT GTG C |
| SbfI-Fy-R | reverse | GGG GGG GGG CCT GCA GGT CAG CTC TTG GAG CCC AGG GTG TC |

**Supplementary Table 2.** Primers and qPCR probes for the assessment of α- and γ-globin mRNA expression

| **Gene** | **Primer direction** | **Primer sequence 5‘ → 3‘** |
| --- | --- | --- |
| α-Globin | forward | CAC GCG CAC AAG CTT CG |
|  | reverse | AGG GTC ACC AGC AGG CAG T |
|  | probe | FAM-TGG ACC CGG TCA ACT TCA AGC TCC T-TAMRA |
| γ-Globin | forward | TGG CAA GAA GGT GCT GAC TTC |
|  | reverse | TCA CTC AGC TGG GCA AAG G |
|  | probe | FAM-TGG GAG ATG CCA TAA AGC ACC TGG-TAMRA |
| GAPDH | forward | AGG GCT GCT TTT AAC TCT GGT AA |
|  | Reverse | CAT GGG TGG AAT CAT ATT GGA AC |
|  | probe | FAM-TGT TGC CAT CAA TGA CCC CTT CAT TG-TAMRA |

**Figure titles and legends**

**Supplementary figure 1. Selection of Fy-K562 clones based on hemoglobin production**

Fy-transduced K562 variants were cultivated on methyl cellulose. Colonies derived from a single cell were picked and plated to test the production of hemoglobin after stimulation with SAHA. HbF levels were determined by FACS analysis using the FMH Quick Quant kit according to the manufacturer´s instructions. Clones showing a level of hemoglobin production higher than 40% were frozen down for further studies.

**Supplementary figure 2. Effect of MIT on the viability of different Fy^b-long^K562 erythroleukemia cell lines.**

**(A)** Effect on the viability of empy vector transduced Fy^b-long^K562 cells **(B)** Effect on the viability of the double transduced Fy^b-long^K562 cells **(C)** Effect on the viability of the locker-miR-26a-5p vector transduced Fy^b-long^K562 cells **(D)** Effect on the viability of the locker-miR-30a-5p vector transduced Fy^b-long^K562 cells. One of four representative experiments is shown.

**Supplementary figure 3. Effect of miRNA down regulation on the production of hemoglobin by benzidine staining.**

**(A-B)** Single transduced Fy^b-long^K562 cells with either the locker-miR-30a-5p **(A)** or the locker-miR-26a-5p vector **(B)** were cultured in four different conditions for 35 days. Every seven days 3 x10^5^ cells were stained with benzidine and cytospins were performed. Bar = 20µm. One of four representative experiments is shown.

**Supplementary figure 4. Effect of miRNA down regulation on the morphology of K562 cells during erythroid differentiation.**

**(A-B)** Single transduced Fy^b-long^K562 cells with either the locker-miR-30a-5p **(A)** or the locker-miR-26a-5p vector **(B)** were cultured in four different conditions for 35 days. Every seven days cytospins of 3 x10^5^ cells were performed and stained with Giemsa solution. Bar = 20µm. One of four representative experiments is shown.

**Supplementary figure 5. Mechanism by which mithramycin A enhances enucleation in double knockdown cells.**

Relative expression of miRNA (fold change) 26a-5p and 30a-5p in locker empty vector (A), locker-miR-26a-5p and locker-miR-30a-5p vectors (B), locker-miR-30a-5p vector (C) and locker-miR-26a-5p vector (D) treated Fy^b-long^K562 cells. The relative expression was measured at day 7 (black bars) and day 21 (green and red bars) in four different conditions. Blue bars in B, C and D indicate comparison with locker empty vector (A) treated control. Relative fold changes in expression (normalized to U6) were calculated by the ΔΔCT method and values are expressed as 2^-ΔΔCT^ (n = 4 independent experiments).

**Supplementary figure 1.**

**
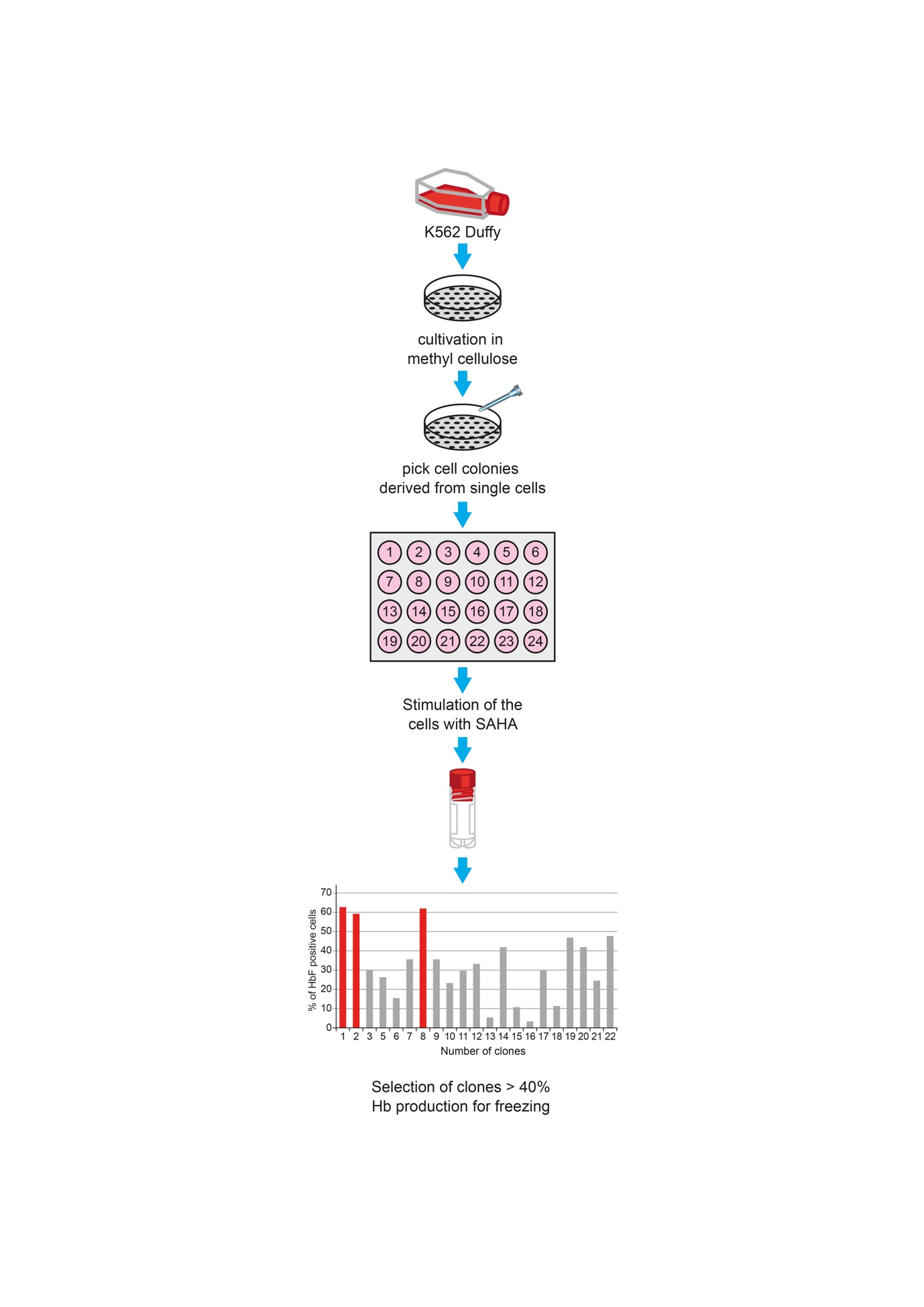
**

**Supplementary figure 2.**

**
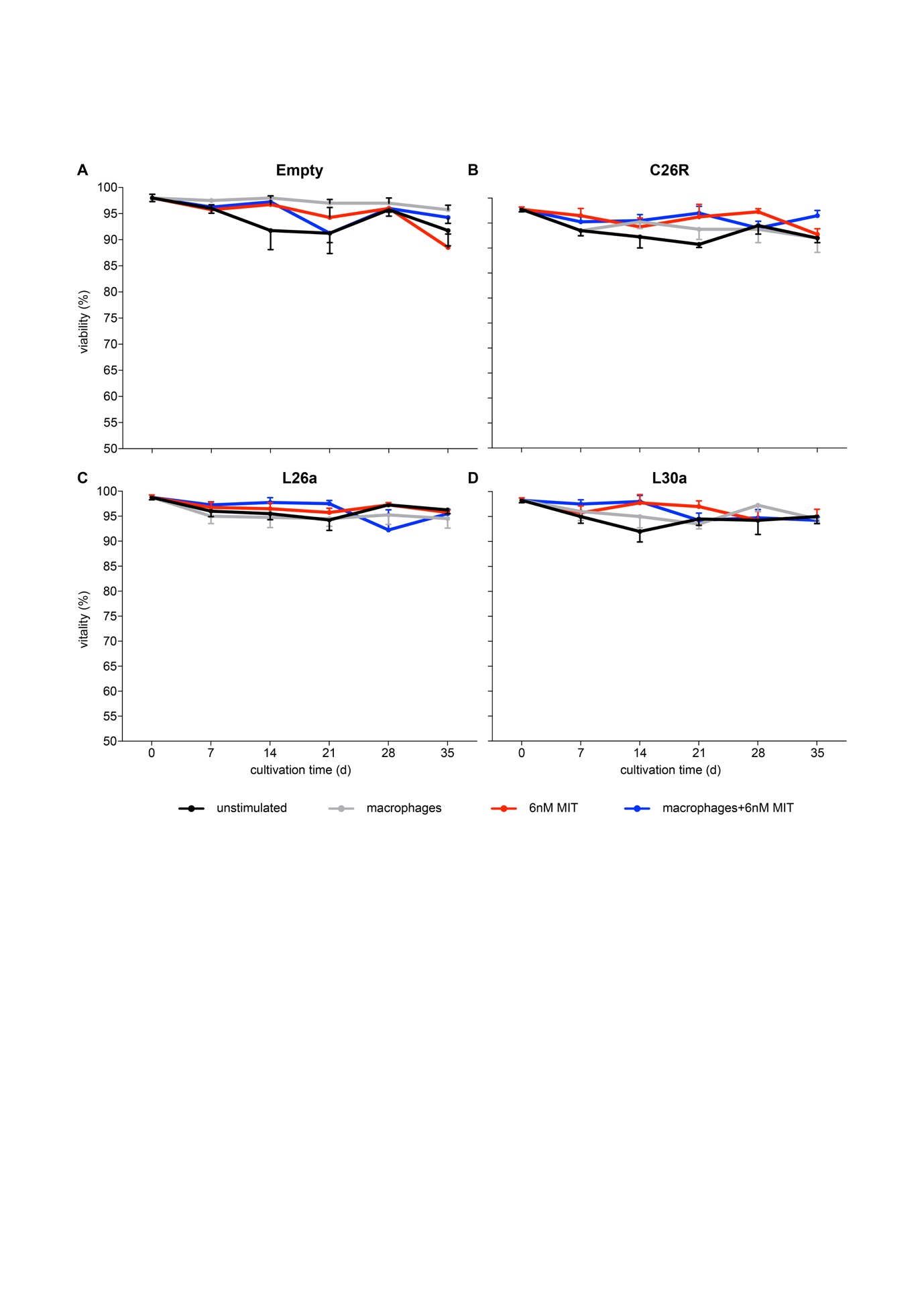
**

**Supplementary figure 3.**

**
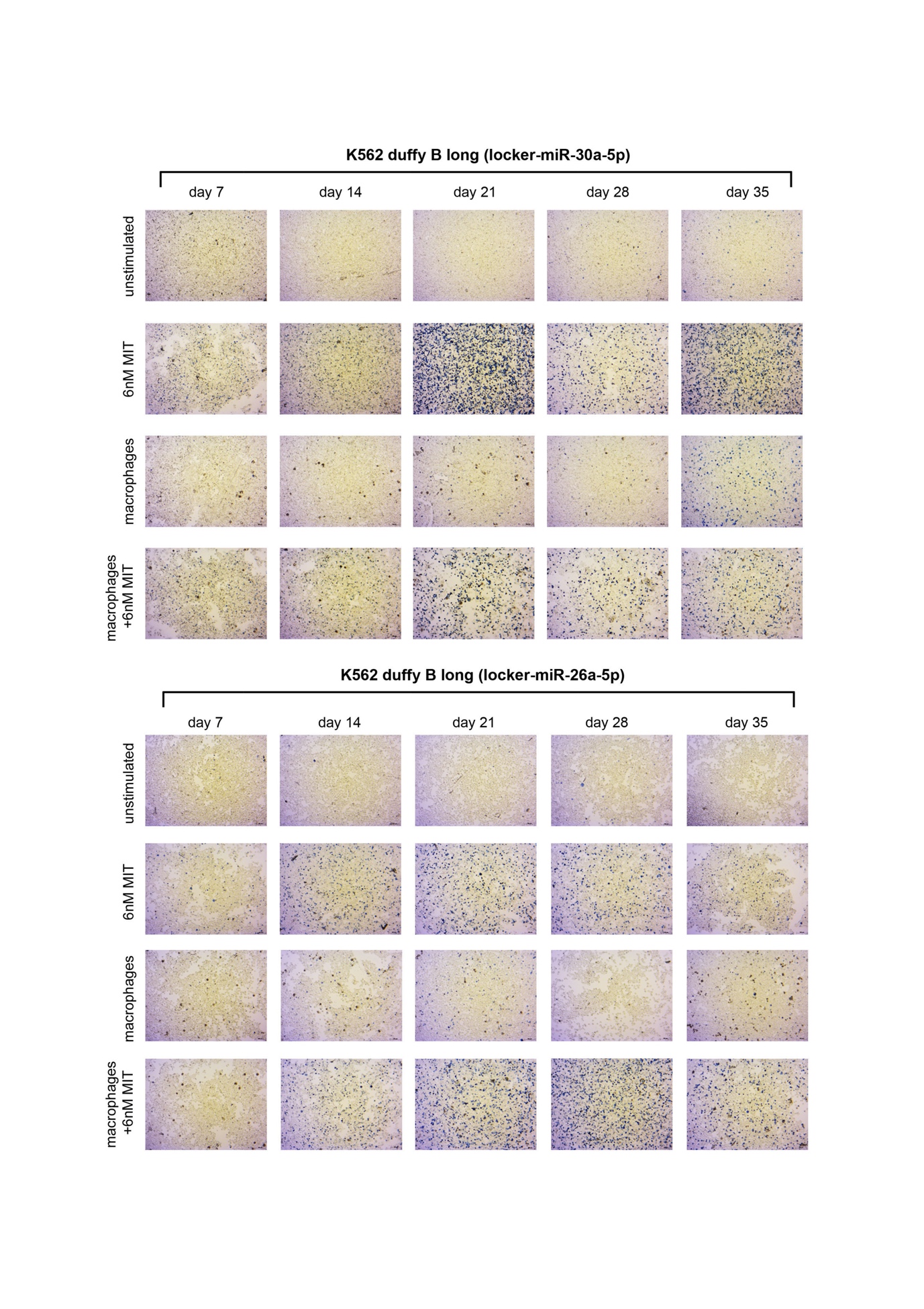
**

**Supplementary figure 4.**

**
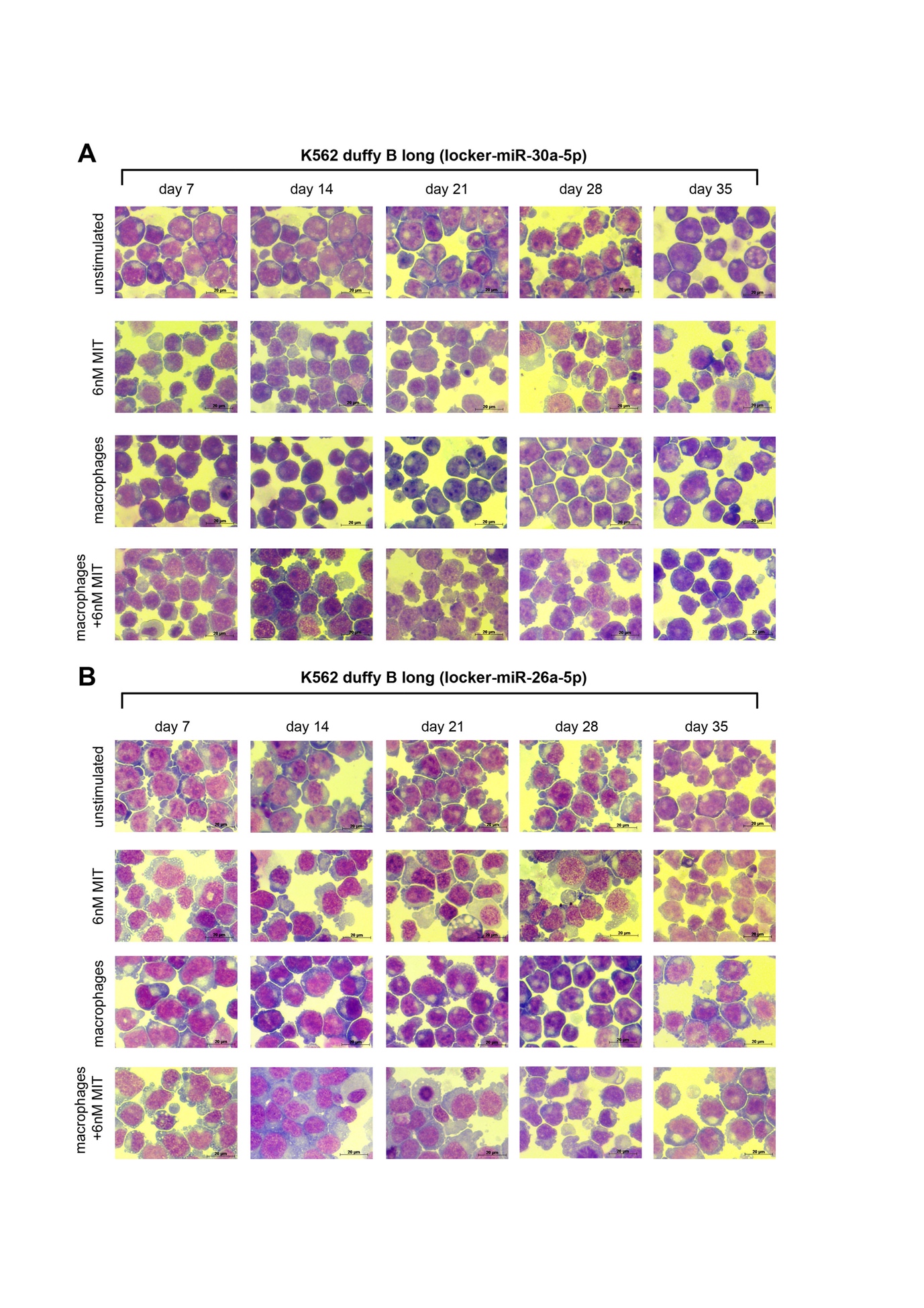
**

**Supplementary figure 5.**

**
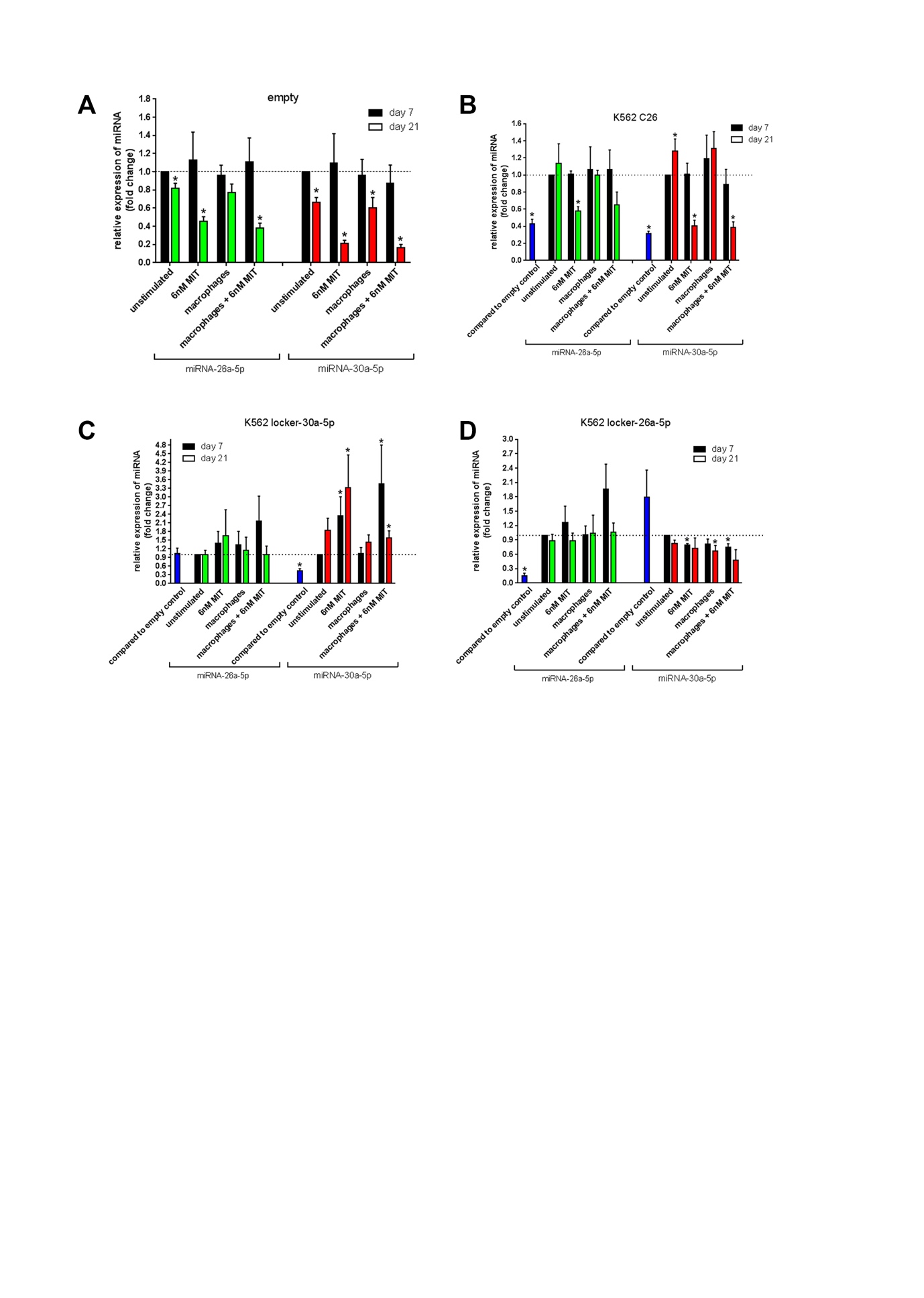
**
